# Simultaneous and synchronous characterization of blood and CSF flow dynamics using multiple Venc PC MRI

**DOI:** 10.1162/imag_a_00521

**Published:** 2025-03-27

**Authors:** Leonardo A. Rivera-Rivera, Tomas Vikner, Chenwei Tang, Laura B. Eisenmenger, Sterling C. Johnson, Kevin M. Johnson

**Affiliations:** Department of Medicine, University of Wisconsin-Madison School of Medicine and Public Health, Madison, WI, United States; Department of Medical Physics, University of Wisconsin-Madison School of Medicine and Public Health, Madison, WI, United States; Department of Radiology, University of Wisconsin-Madison School of Medicine and Public Health, Madison, WI, United States; Department of Diagnostics and Intervention, Umeå University, Umeå, Sweden

**Keywords:** interleaved, flow encoding, neurofluids, phase contrast MRI, multi-venc

## Abstract

Neurofluid dynamics are crucial for maintaining brain homeostasis and facilitating the clearance of brain metabolites through the coupling of arterial and venous blood with cerebrospinal fluid (CSF). Two-dimensional phase contrast (PC) magnetic resonance imaging (MRI) is frequently used to study neurofluids; however, separate examinations are typically required for assessing blood and CSF flow, which can confound analyses due to asynchronous physiological measurements. To enable simultaneous assessment of neurofluid dynamics, we describe and evaluate a 2D PC MRI approach in human participant experiments. An interleaved multi-point velocity encoding scheme was integrated into a 2D golden angle spiral PC MRI scan to facilitate synchronous characterization of neurofluids. Two multi-point schemes, including interleaved dual-venc (DV) and triple-venc (MV) scans, were evaluated and compared with standard asynchronous single-venc (SV) scans. Data and repeated scans were collected on a clinical 3.0T scanner at the level of the C1/C2 vertebrae in 10 human participants. From cardiac-resolved images, the relationship between net blood flow and CSF flow pulsatile volume change was characterized using regression modeling. Temporal lags between cardiac-driven arterial blood (vertebral arteries (VAs) and internal carotid arteries (ICAs)) and spinal canal (SC) CSF were estimated with cross-correlation. SV, DV, and MV flow mean, range, and volume changes were studied and compared using linear mixed effect models, intraclass correlation coefficients, Bland–Altman, and Pearson correlations. A strong relationship was measured between net blood flow and CSF flow pulsatile volume change from SV (R^2^= 0.71, P = 0.002), DV (R^2^= 0.70, P = 0.003), and MV (R^2^= 0.78, P < 0.001) scans. SC-VAs temporal lags were statistically longer than SC-ICAs lags across all scans (P < 0.001 for SV, DV, and MV). Bland–Altman analyses and repeatability coefficients indicated that DV and MV scans had the highest repeatability. MV scans generally underestimated SC CSF flow markers relative to SV and DV scans. A more pronounced flow offset in venous measures was identified between SV scans and the DV and MV scans. In conclusion, this study introduced a method for simultaneous imaging of cranio-spinal arterial, venous, and CSF flow, enabling the synchronous assessment of neurofluid dynamics. The results indicated that interleaved DV and MV scans could improve the evaluation of neurofluid coupling compared with asynchronous SV scans.

## 1 Introduction

Cerebral blood flow (CBF), cerebrospinal fluid (CSF), and interstitial fluid (ISF) are essential neurofluids that maintain and regulate brain homeostasis (Menéndez González, 2023). CBF both delivers and drains substrates supporting metabolic functions (Claassen et al., 2021). CSF provides hydromechanical protection, regulates ISF homeostasis, and drains soluble metabolic waste from the brain (Sakka et al., 2011). The fixed volume of the cranium enforces a tight dynamic coupling between CBF and CSF with modulations from cardiac and respiratory forces and neuronal activity (Wang et al., 2022). Disruptions to this coupling in conditions such as traumatic brain injury, hydrocephalus, and stroke can lead to increased intracranial pressure and other serious complications (Bothwell et al., 2019). More recently, impaired CSF circulation has been implicated in the development of proteinopathies, such as beta-amyloid plaques, due to the failure of waste clearance (e.g., glymphatic clearance) (Carlstrom et al., 2022). To address the current clinical needs and understand normal and impaired neurofluid homeostasis, methods are needed to evaluate both CSF and CBF dynamics.

Magnetic resonance imaging (MRI) provides various techniques to evaluate neurofluids (Agarwal et al., 2024) including hemodynamics with BOLD-fMRI (Fultz et al., 2019), transport with intrathecal and intravenous injections (van Osch et al., 2024), and CSF motility with diffusion-based methods (Wright et al., 2024). MRI also provides phase contrast (PC) velocimetry, the standard for clinical blood and CSF flow imaging (Alperin, 2020;Balédent et al., 2004;Owashi et al., 2023). While cardiac phase-resolved 2D PC MRI is a long standing clinical standard, recent technology advances have provided the potential for real-time imaging to measure flow-related cardiac and respiratory effects (Aktas et al., 2019;Laganà et al., 2022;Liu et al., 2022;Owashi et al., 2023;Spijkerman et al., 2019;Töger et al., 2022), and 3D time-resolved (4D-Flow) imaging of both blood flow (Roberts et al., 2023;Söderström et al., 2025;Wåhlin et al., 2022) and CSF flow (Rivera-Rivera et al., 2024;Vikner et al., 2024). Unfortunately, PC MRI requires the selection of a velocity-sensitizing parameter (venc) which must be set based on the fluid of interest. Consequently, measures of CSF, arterial, and venous flow are acquired asynchronously in separate scans and the comparison of these measures is affected by variations in heart rate, respiration, and the repeatability of external challenges (e.g., respiratory maneuvers, exercise, neurostimulation) (Laganà et al., 2022;Morgan et al., 2023;Sakhare et al., 2019). This physiological variability can confound the evaluation and repeatability of PC MRI-based neurofluid coupling measures (Alperin, 2020;Alperin et al., 2021).

Separate PC MRI scans are often acquired with a venc to match the significantly different velocities of arterial (<150 cm/s), venous (<40 cm/s), and CSF (<10 cm/s) flows (Rivera-Rivera et al., 2024). The venc is the maximum velocity that can be detected without phase aliasing and is roughly proportional to velocity noise (Vnoise ~ venc/SNR) (Nayak et al., 2015). Ideally, synchronous neurofluid measurements are acquired from a single PC MRI scan that interleaves velocity encodings (i.e., multi-vencs). Imaging arterial and venous flow is achievable through dual-venc approaches (Aristova et al., 2019;Schnell et al., 2017), which typically use high-venc data to phase unwrap low-venc data. This provides a boost in velocity-to-noise ratio (VNR), but the velocity dynamic range is still limited to arterial and venous blood flow, missing the slower CSF flow. Such dual-venc PC MRI approaches also do not interleave velocity encodings. Some progress has been made in simultaneous multi-venc acquisitions using multi-slice (e.g., SMS) and interleaved encoding (Park et al., 2020). However, these methods can be limited by the restricted selection of low venc due to signal loss from prolonged TE and the difficulty in separating inter-venc information due to insufficient sensitivity variations in the venc direction.

This study develops and investigates a multi-venc 2D PC MRI approach for the synchronous characterization of cranio-spinal neurofluid dynamics, specifically exploring imaging with a wide venc range sufficient to image both CSF and blood flow. The proposed approach consists of an interleaved multi-venc acquisition with a minimal time first moment gradient design and golden angle spiral sampling. These features aim to reduce higher-order flow effects, allow for flexible selection of vencs, achieve short echo times, and support retrospective cardiac-resolved reconstructions. Experiments were conducted on 10 healthy human participants using 2 different multi-venc protocols: a dual-venc scan and a triple-venc scan (referred to as multi-venc from here on). These were compared against asynchronous standard single-venc 2D PC MRI scans. Test–retest repeatability experiments were also performed. Cardiac-driven neurofluid coupling was studied, including blood flow (arterial and venous) and CSF flow pulsatile volume changes and temporal dynamics.

## 2 Methods

### 2.1 Interleaved multiple venc flow encoding

One-sided, one directional flow encoding perpendicular to the slice direction was utilized. Flow encoding bipolar gradients were independently designed for multiple vencs with each collected with the same echo time (TE), set based on the flow encoding with the largest 1^st^gradient moment (i.e., lowest venc flow encode). Each flow encoding was independently designed to be time minimal using minimum-time optimization to reduce sensitivity to higher-order flow effects. Flow encodings were collected in an interleaved fashion with identical k-space sampling. K-space was collected continuously with a center out 2D golden angle spiral trajectory to enable flexible and efficient data collection with short echo times and retrospective reconstruction. A measurement block consisted of a flow compensated encode, followed by high- to low-venc encodes.

**Fig. 1.**
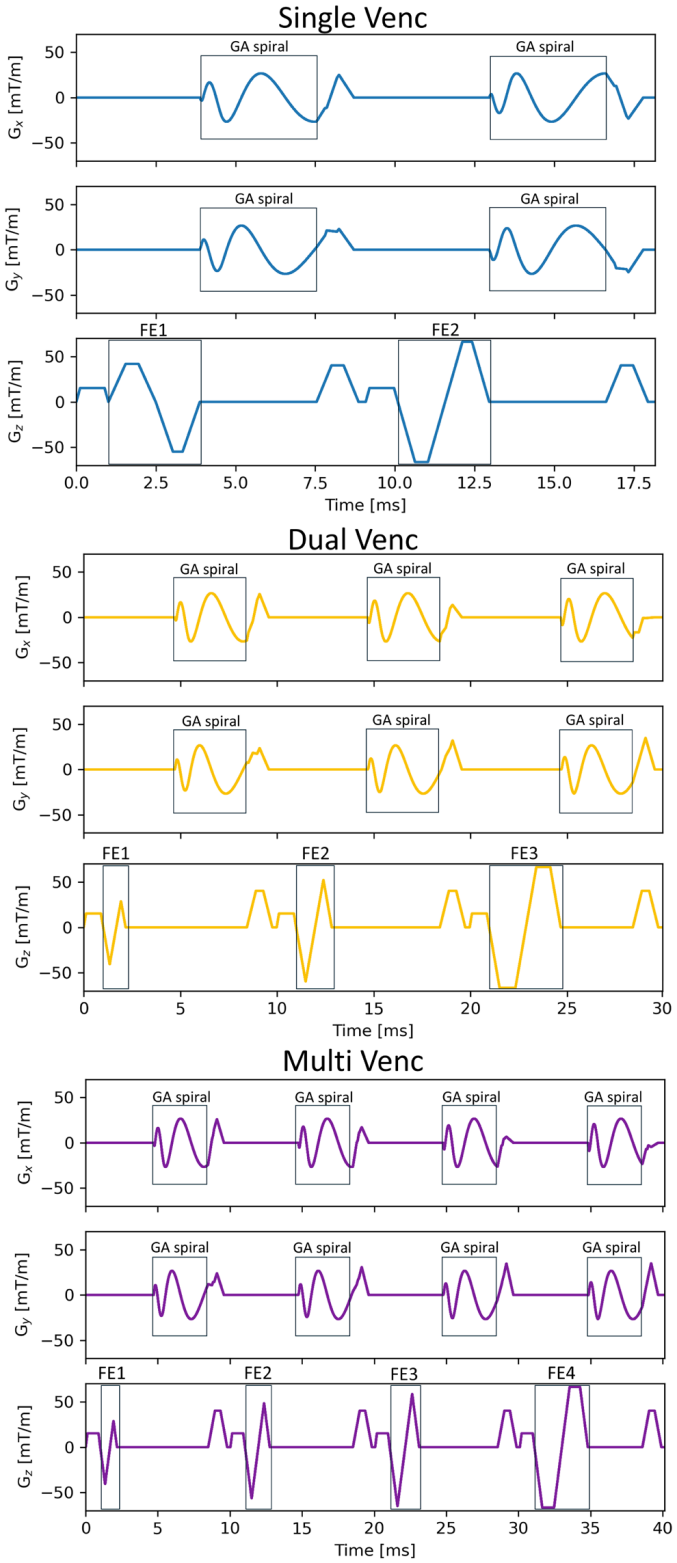
Pulse sequence diagrams of the single-venc (top) and the proposed interleaved dual-venc (middle) and multiple-venc (bottom) 2D PC MRI acquisitions. (Top) The single-venc acquisition (example shown for venc = 8 cm/s) used to compare the proposed dual- and multi-venc approaches utilized two-side balanced flow encoding (FE) to achieve the shortest possible TEs and TRs, and golden angle (GA) spiral sampling. Single directional dual- and multi-venc flow encoding was enabled by designing different sets of flow encoding bipolar gradients applied along the z-axis which were interleaved during acquisition to capture similar physiology across different venc settings and enable synchronous characterization of neurofluid dynamics. For each FE step, the 1^st^gradient moment was recalculated to be time minimal (i.e., minimum-time optimization) to reduce higher-order flow effects. The flow encoding module is followed by center out GA 2D spiral sampling to enable flexible reconstruction support and short echo times. In the dual-venc diagram (middle), three flow encodes are illustrated including a flow compensated reference encode (FE1), and high-venc (75 cm/s) (FE2) and low-venc (8 cm/s) (FE3) encodes. In the multi-venc diagram (bottom), four flow encodes are illustrated including a flow compensated reference encode (FE1), and high-venc (100 cm/s) (FE2), medium-venc (50 cm/s) (FE3), and low-venc (8 cm/s) (FE4) encodes.

In the study, two multiple venc acquisitions were evaluated: (1) a three-point encoding dual-venc scan with vencs of 75 and 8 cm/s and (2) a four-point encoding triple-venc (multi-venc) scan with vencs of 100, 50, and 8 cm/s. These acquisitions explored a balanced intermediate venc (75 cm/s) and high and lower vencs (100 and 50 cm/s) for arterial and venous blood flow, and a much lower venc for spinal canal CSF flow (8 cm/s), based on expected flow velocities (Balédent et al., 2004). Interleaved dual-venc and multi-venc scans were compared against standard asynchronous single-venc 2D PC MRI scans in human experiments. Separate single-venc scans were acquired with vencs of 75 and 8 cm/s, with the same golden angle spiral sampling. Single-venc scans used two-side (e.g., +, -) balanced flow encoding to achieve the shortest possible TEs and TRs (Pelc et al., 1991).Figure 1shows pulse sequence diagrams for the single-venc (top) and the proposed interleaved dual-venc (middle) and multiple-venc (bottom) 2D PC MRI flow encoding schemes.

### 2.2 Study participants

MRI data were collected in healthy human participants (N = 10, age range 22–49 y, mean 28 ± 7 y, 6 female). Participants underwent a single MRI session including T2-weighted and 3D time-of-flight angiography scans for localization and 2D PC scans. The University of Wisconsin Institutional Review Board approved all study procedures and protocols following the policies and guidance established by the campus Human Research Protection Program. Each study participant signed a written informed consent before participation.

### 2.3 MRI protocol

MRI data were acquired on a clinical 3.0T system (SIGNA Premier, GE Healthcare) (maximum gradient strength = 80 mT/m, maximum slew rate = 200 T/m/s) using a 48-channel head-coil (GE Healthcare). Sagittal 3D CUBE T2-weighted images were acquired with an imaging volume of 25 x 25 x 20.5 cm^3^and 1 x 1 x 1.6 mm^3^spatial resolution. Axial 3D time-of-flight images were collected with an imaging volume of 24 x 24 x 4 cm^3^and 1 x 1 x 1 mm^3^spatial resolution. 2D PC scans were prescribed at the level of the C1/C2 vertebrae using the T2-weighted and time-of-flight angiographic images to capture arterial inflow (internal carotid arteries (ICAs) and vertebral arteries (VAs)), venous outflow (internal jugular veins (IJVs)), and bidirectional CSF flow (spinal canal (SC)) within the same imaging slice. All 2D PC scans were prescribed axially with a field of view of 22 x 22 cm^2^, slice thickness = 4 mm, in-plane resolution = 0.68 x 0.68 mm^2^, flip angle = 8°, and receiver bandwidth = ± 125 kHz. Individual high and low single-venc scans were acquired with venc = 75 cm/s, TR/TE = 8.0/2.1 ms, scan time = 53 s, and venc = 8 cm/s, TR/TE = 9.2/3.4 ms, scan time = 61 s. Dual-venc scans parameters included venc = 75 and 8 cm/s, TR/TE = 10.1/4.2 ms, and scan time = 61 s. Multi-venc scans parameters included venc = 100, 50, and 8 cm/s, TR/TE = 10.2/4.2 ms, and scan time = 61 s. The number of spiral arms and k-space oversampling factors were (3300, 34), (2000, 21), and (1500, 16) for single-, dual-, and multi-venc scans, respectively. With the ratio of the number of arms and acceleration fixed, the spiral readout window was the same for all scans with a sampling window of ~3.7 msec. These values were selected to achieve a scan time of approximately 1 min while keeping secondary effects from off-resonance and T2* decay similar across all scans. The theoretical temporal resolution for single-venc scans with balanced encoding was ~18 ms (2 TRs). For dual- and multi-venc scans with referenced encoding, the theoretical temporal resolution was ~10 ms (1 TR), as each venc is achieved per bipolar encode and encodes can be split during image reconstruction (Rivera-Rivera et al., 2021).

All 2D PC scans were repeated for test–retest evaluation. Repeated scans were performed with the participant in the same position and used the same scan planning plane prescription. The order of scan acquisition was single-venc (75 cm/s), single-venc (8 cm/s), two times dual-venc, single-venc (75 cm/s), single-venc (8 cm/s), and two times multi-venc scans. The time between single-venc (75 cm/s) and single-venc (8 cm/s) scan repeats was ~183 s and ~175 s, respectively. Dual- and multi-venc repeats were acquired back-to-back. Cardiac triggers were collected for each participant from a photoplethysmogram on a pulse oximeter (GE Healthcare) worn on the participant’s finger during the MRI examination.

### 2.4 Image reconstruction, post-processing, and flow quantification

Magnitude and velocity images were retrospectively reconstructed from 2D PC MRI data to 30 cardiac phases using cardiac triggers and SENSE with a low rank temporal constraint with adaptative thresholding and block size = 8 x 8 (Rivera-Rivera et al., 2021). The number of spiral arms per reconstructed cardiac phase was 110, 67, and 50, for single-, dual-, and multi-venc scans, respectively. Thus, the undersampling factor, approximated as the ratio of the number of times k-space was oversampled over the number of cardiac phases reconstructed, was 1.13, 0.70, and 0.50, respectively. Data corrections were performed including 2^nd^order polynomial fit of static tissue phase to remove background phase offsets, and single step Laplacian for phase anti-aliasing (Loecher et al., 2016). For multi-venc scans, the high-venc encode (100 cm/s) was used to phase unwrap the middle-venc encode (50 cm/s) used in comparisons. Flow measurements were performed similar to other 2D PC MRI studies (Wåhlin et al., 2012) using FIJI ImageJ (v1.54f) (Schindelin et al., 2012). Images were visually inspected for motion corruption. Flow processing consisted of semi-automatic segmentation of vessels in the single-venc scans (venc = 75 cm/s) using time-averaged magnitude and angiographic images, and of SC from single-venc scans with venc = 8 cm/s using the standard deviation of the velocity images. Cardiac-resolved flow waveforms were estimated from the segmented regions of interest (ROIs) area and spatial mean in the velocity cardiac series. The same set of ROIs was used for extraction of flow from dual-venc and multi-venc scans after rigid re-adjustment for inter-scan motion. All ROIs were visually inspected to verify and correct alignment of the vessel of interest and spinal canal across the different scans. From visual inspection, no changes were perceived in vessel or spinal canal size across scans. ROIs included left and right ICAs, VAs, IJVs, and SC. Flow parameters derived from the extracted cardiac flow waveforms included mean flow rate (mean), amplitude of the flow rate curve (range), and pulsatile volume change (ΔV) defined as the range of the cumulative integral of the demeaned flow waveform, as described inWåhlin et al. (2012). The pulsatile volume change ΔV is akin to stroke volume, reflecting features of the cardiac dynamics. Total cerebral blood flow (tCBF) was defined as the addition of ICAs and VAs flow, while total jugular blood flow (tJBF) was the addition of left and right IJVs.

### 2.5 Statistical analyses

Regression analysis was used to study the relationship between net blood flow (ΔV (tCBF- tJBF)) and CSF flow (ΔV SC) pulsatile volume change, as it has been proposed that CSF pulsations follow net transcranial pulsatile blood flow (Alperin, 2020). Time lags between ICAs and VAs blood flow, and SC CSF flow were estimated using cross-correlation (MATLAB R2019b*xcorr()*function) on interpolated flow curves (500 time points using MATLAB R2019b*interp1()*function with linear interpolation). Before measuring time lags, flow curves from single-venc scans (75 cm/s and 8 cm/s) were resampled to the same cardiac cycle length, to reduce effects from heart rate variability from separate scans. Flow curves were cardiac cycle referenced based on actual encoding time. For example, flow profiles from the dual-venc high-venc encode were shifted by 1TR, and the dual-venc low-venc encode by 2TR. For comparison and visualization purposes, cardiac-resolved flow curves were demeaned. Linear mixed effects (LME) models were used to determine fixed effects from scan type (i.e., single-venc, dual-venc, multi-venc) (categorical predictor) and heart rate (continuous predictor), and random effects from intercepts grouped by participants for flow mean, range, and ΔV measurements. For the categorical predictors (scan type), the effects were relative to the single-venc scans (the reference category from the model setup). For continuous predictor (heart rate), the effect was the slope of the relationship between heart rate and the flow parameter of interest. To determine differences across scan type, pair-wise comparisons were performed using an F-test on the fixed-effects estimated coefficients and confidence intervals. Quantile–quantile plots of LME models residuals were used to verify assumptions of normality. The random effects accounted for the variability between participants and repeated measures, providing insight into whether flow parameter differences were primarily between participants (e.g., physiological differences) or within participants (e.g., more likely technical variability). To determine how much of the observable variability in flow markers was between-participant as opposed to within-participant, intra-class correlation coefficients were estimated using$\frac{\sigma_{volunteer}^{2}}{\sigma_{volunteer}^{2}+\sigma_{error}^{2}}$, where$\sigma_{volunteer}^{2}$and$\sigma_{error}^{2}$are the variances due to participant and standard error from random effects. Bland–Altman analyses were performed to estimate mean differences, agreement intervals, bias, and repeatability coefficients (RPC) between markers for repeated scans and across scan types (Bland & Altman, 1999). RPC was defined as (1.96σ)*100, whereσis the standard deviation of the differences (d = y1−y2) each expressed as a proportion of their mean (m = (y1 + y2)/2) (Bland & Altman, 1986). Pearson correlation coefficients were estimated across scan types (e.g., single-venc vs. dual-venc), and between repeated scans (e.g., dual-venc #1 vs. dual-venc #2). Statistical analyses were performed in MATLAB. P < 0.05 was set as the threshold for statistical significance.

## 3 Results

Images were successfully collected in all participants. The mean heart rate across all scans was 65 ± 11 bpm (range 41–83 bpm), corresponding to a temporal resolution of ~31 ms in the reconstructed images. A summary of heart rate differences across the different scans for each participant is included inSupplemental Figure 1. No evidence of bulk motion was identified in any set of images.

### 3.1 Exemplary images and flow profiles

**Fig. 2.**
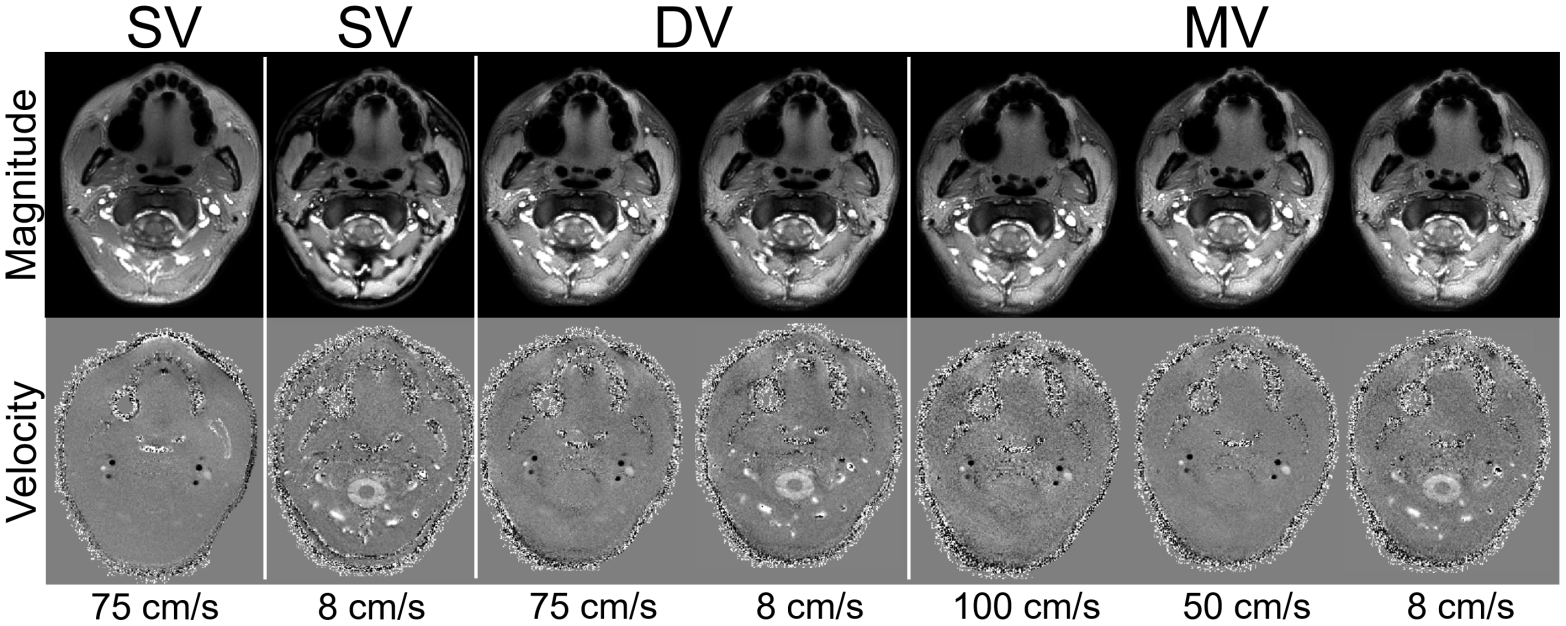
Example of magnitude (top) and velocity (bottom) images reconstructed from four 2D PC MRI scans using different flow encoding schemes in a study participant. (left to right) Single-venc (SV) scans (75 and 8 cm/s) acquired separately, and the proposed interleaved dual-venc (DV) (75 and 8 cm/s) and multi-venc (MV) (100, 50, and 8 cm/s) scans. Good subjective image quality and conspicuous depiction of the internal carotid arteries, vertebral arteries, internal jugular veins, and spinal canal were observed across scans including the DV and MV scans despite their longer echo times (TE) (SV TEs = 2.1 and 3.4 ms for vencs of 75 and 8 cm/s; DV and MV both with TE = 4.2 ms) and undersampling factors (1.13, 0.70, and 0.50 for SV, DV, and MV). All scans had a similar acquisition time ~1 min.

Figure 2shows 2D PC MRI magnitude and velocity images from one participant including single-venc (both high- and low-venc acquisitions), and the interleaved dual-venc and multi-venc scans. Good subjective image quality was observed across scan types. Cardiac-resolved flow profiles in ICAs, VAs, IJVs, and SC for two participants are included inFigure 3. Overall cardiac waveform shape depiction was similar across scan types; however, single-venc flow profiles showed a constant offset which was more noticeable on venous measurements. This offset was partially attributed to incomplete removal of the background phase in the single-venc scans as observed inSupplemental Figure 2.

**Fig. 3.**
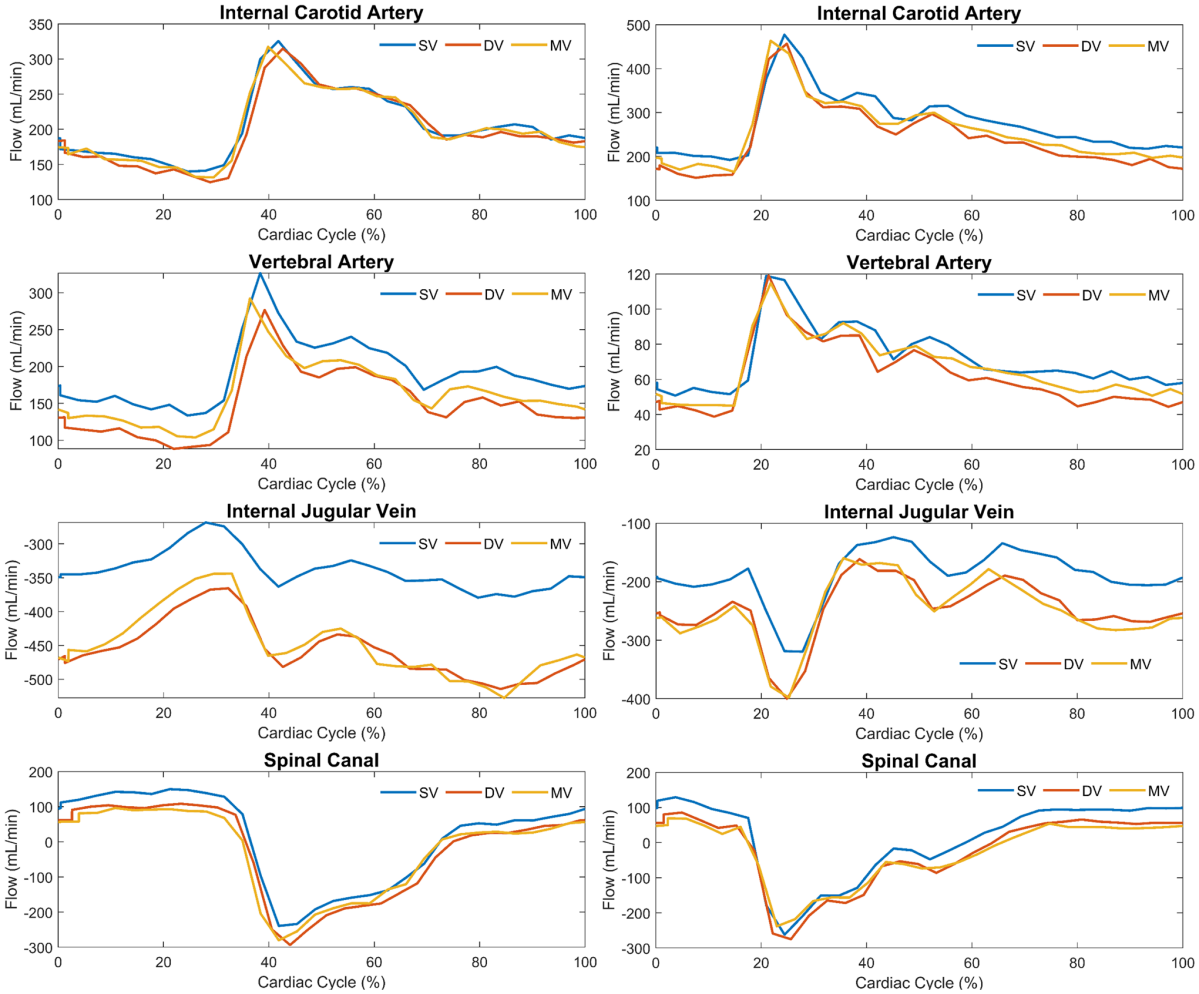
Example of cardiac-resolved flow profiles derived from single-venc (SV), dual-venc (DV), and multi-venc (MV) 2D PC MRI scans on two study participants (left and right columns). Flow profiles are shown for the right internal carotid artery, vertebral artery, internal jugular vein, and the spinal canal (top to bottom). Overall, cardiac flow profiles were similar across scans. A constant flow offset was observed in the SV scans more noticeable in the internal jugular vein than in the DV and MV scans, partially attributed to incomplete removal of background phase in the SV scans (Supplemental Fig. 2).

### 3.2 CBF and CSF cardiac coupling

Linear regression models assessing the relationship between net blood flow and CSF flow pulsatile volume change (ΔV) are shown inFigure 4. A strong relationship between net blood flow (tCBF – tJBF) ΔV and CSF flow (SC) ΔV was observed across all scans (R^2^≥ 0.70). Multi-venc scans coefficient of determination was highest with R^2^= 0.78 (P < 0.001), compared with single-venc R^2^= 0.71 (P = 0.002) and dual-venc R^2^= 0.70 (P = 0.003) scans. The average differences between net blood flow ΔV and CSF flow ΔV were 73 ± 188 µL, 73 ± 236 µL, and 140 ± 261 µL for single-, dual-, and multi-venc scans.

**Fig. 4.**
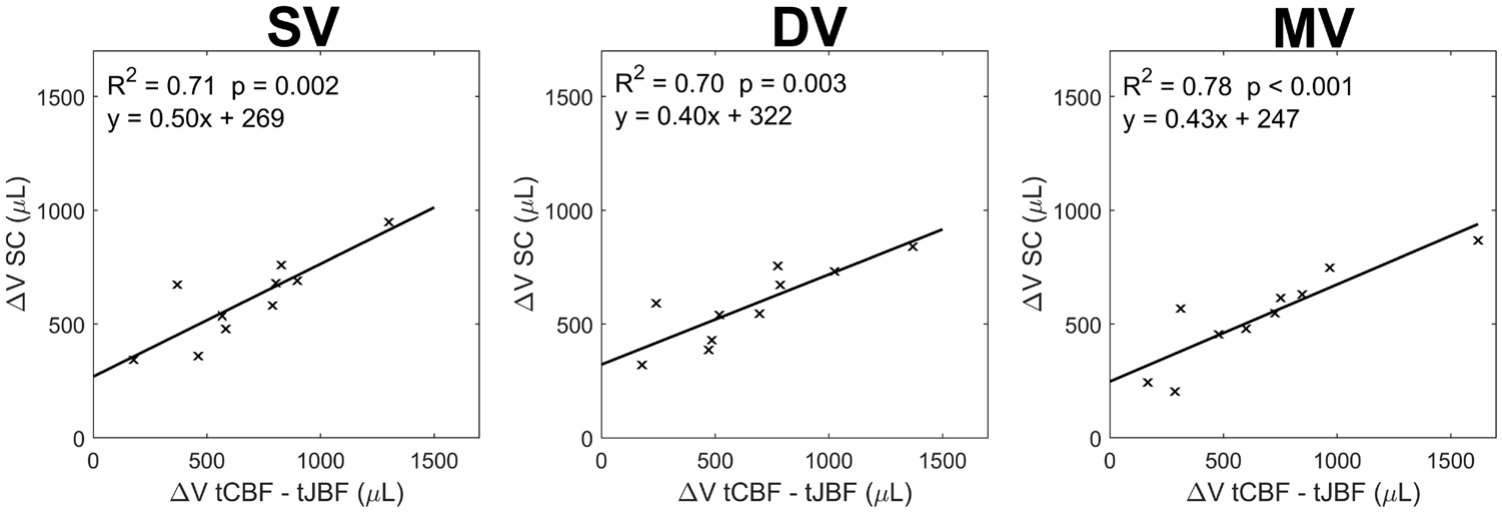
Participants (N = 10) pulsatile volume change (ΔV) relationship between net blood flow (total cerebral blood flow (tCBF) – total jugular blood flow (tJBF)) and CSF flow (spinal canal (SC) CSF) for each scan type: single-venc (SV) (from separate acquisitions) (left), interleaved dual-venc (DV) (middle), and interleaved multi-venc (MV) (right). A strong correlation between net blood flow and CSF flow ΔV was observed across all scans (R^2^≥ 0.70).

Dual-venc demeaned cardiac-resolved flow profiles in the ICAs, VAs, and SC for all participants including repeated scans are shown inFigure 5for comparison. Visual inspection shows the lower pulsatile flow rates across the VAs. Similar waveform shapes were observed for ICAs, VAs, and SC flow curves derived from single-venc and multi-venc scans (Supplemental Figs. 3 and 4). Cross-correlation analyses quantifying SC CSF flow lags with respect to ICAs (avg. ~5 ms) and VAs (avg. ~19 ms) blood flow are summarized inFigure 6. The SC-VAs lag was longer than SC-ICAs in all scans (P < 0.001 for single-, dual-, and multi-venc). Multi-venc lags were shorter than single-venc (SC-ICAs P = 0.012; SC-VAs P = 0.001) and dual-venc (SC-ICAs P = 0.007; SC-VAs P = 0.001) scans.

**Fig. 5.**
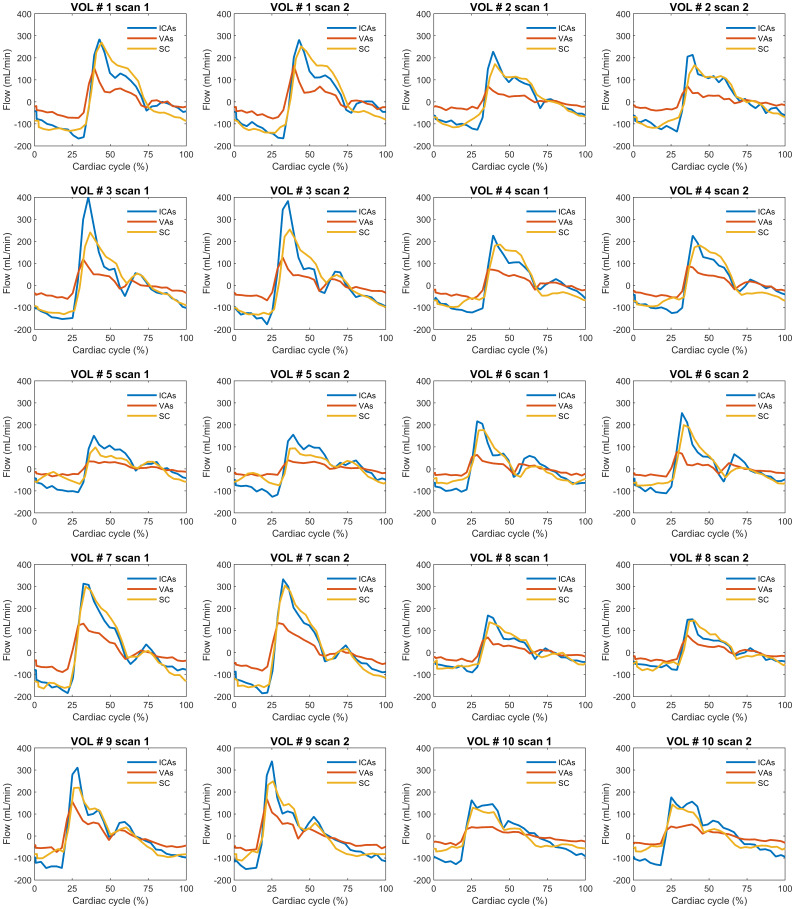
Dual-venc cardiac-resolved internal carotid arteries (ICAs) and vertebral arteries (VAs) blood flow and spinal canal (SC) CSF flow profiles (demeaned) for all 10 participants and repeated scans used to study the temporal lag between ICAs, VAs inflow, and SC flow. Note that arterial (ICAs, VAs) blood and SC CSF flow in opposite directions through the imaging slice; hence, the CSF waveforms were inverted to facilitate comparisons. Flow profiles measured from single-venc and multi-venc scans are included inSupplemental Figures 3 and 4.

**Fig. 6.**
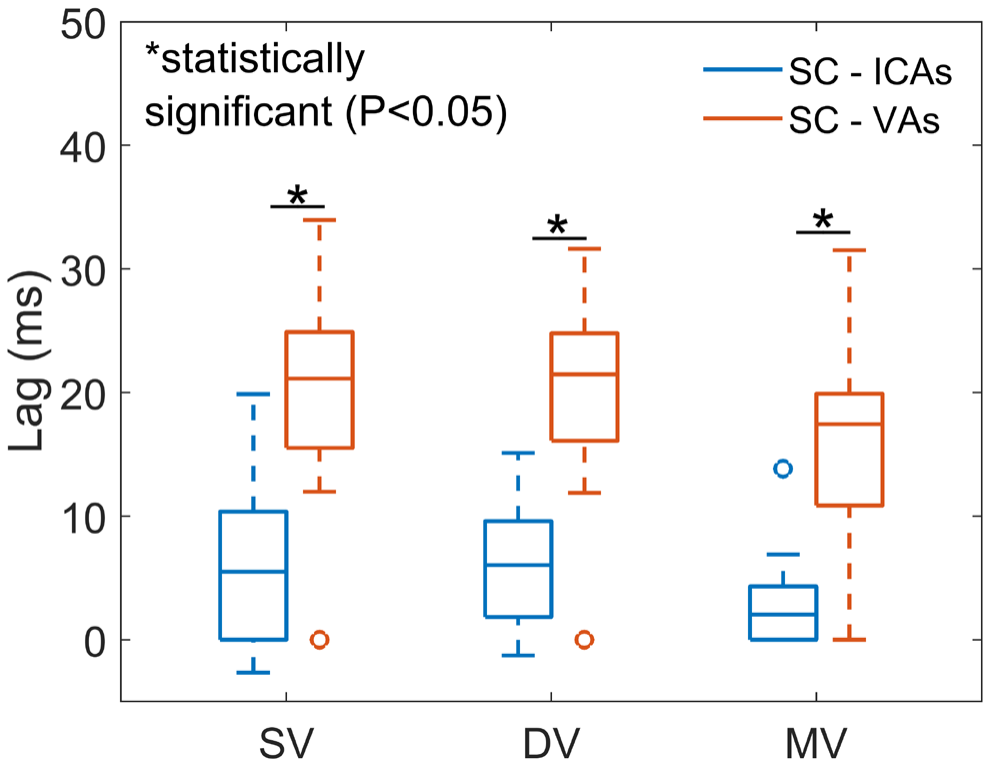
Summary from 10 participants of cardiac-resolved lags between spinal canal CSF flow and internal carotid arteries (ICAs) (blue box) and vertebral arteries (VAs) (orange box) blood flow waveforms for different scans including single-venc (SV), dual-venc (DV), and multi-venc (MV). Lags from SV scans were estimated from separate high- and low-venc acquisitions. The mean SC-ICAs lags were 6 ± 7 ms, 6 ± 5 ms, and 3 ± 5 ms for SV, DV, and MV scans. The SC-VAs lags were longer: 20 ± 10 ms, 20 ± 9 ms, and 16 ± 9 ms. For all encoding schemes, SC-VAs lags were statistically longer than SC-ICAs lags (P < 0.001 for SV, DV, and MV). SC-ICAs and SC-VAs lags for MV were significantly shorter than SV (P = 0.012, P = 0.001) and DV (P = 0.007, P = 0.001), respectively. VA-ICAs lags were 13 ± 6 ms, 14 ± 5 ms, and 13 ± 5 ms indicating cardiac blood flow from the VAs precedes ICAs at the level of the C1/C2 vertebrae in the studied group.

### 3.3 Quantitative comparisons across flow encoding schemes

The average flow range of CSF in the spinal canal was 312 ± 85 mL/min, 300 ± 91 mL/min, and 273 ± 90 mL/min, while ΔV values were 605 ± 186 µL, 581 ± 170 µL, and 536 ± 205 µL from single-venc, dual-venc, and multi-venc scans, respectively. The mean flow of tCBF was 634 ± 112 mL/min, 575 ± 119 mL/min, and 582 ± 107 mL/min, while tCBF flow range was 522 ± 143 mL/min, 511 ± 145 mL/min, and 507 ± 150 mL/min from single-venc, dual-venc, and multi-venc scans, respectively. Furthermore, tCBF average ΔV values were 844 ± 245 µL (single-venc), 856 ± 262 µL (dual-venc), 870 ± 325 µL (multi-venc). A complete summary of flow means, range, and ΔV averages for all the participants in measured ROIs is included inTable 1.

**Table 1.** Summary of flow mean, range, and volume change (ΔV) measurements across participants (N = 10) for blood and CSF.

	Flow (mL/min)			Flow range (mL/min)			ΔV (µL)		
	SV	DV	MV	SV	DV	MV	SV	DV	MV
ICA (R)	241 ± 33	225 ± 44	227 ± 38	204 ± 59	206 ± 64	204 ± 62	330 ± 104	337 ± 117	347 ± 134
ICA (L)	215 ± 48	203 ± 47	204 ± 44	190 ± 53	181 ± 50	185 ± 58	292 ± 78	288 ± 79	298 ± 101
VA (R)	86 ± 28	70 ± 26	70 ± 26	70 ± 37	69 ± 37	63 ± 36	111 ± 72	115 ± 70	112 ± 81
VA (L)	95 ± 46	77 ± 35	81 ± 36	84 ± 46	83 ± 46	77 ± 42	118 ± 45	121 ± 44	118 ± 45
**tCBF**	634 ± 112	575 ± 119	582 ± 107	522 ± 143	511 ± 145	507 ± 150	844 ± 245	856 ± 262	870 ± 325
IJV (R)	259 ± 97	307 ± 144	324 ± 113	148 ± 81	175 ± 76	182 ± 92	249 ± 156	289 ± 163	325 ± 189
IJV (L)	142 ± 86	182 ± 107	179 ± 96	69 ± 44	86 ± 50	86 ± 46	124 ± 98	153 ± 115	156 ± 109
**tJBF**	402 ± 147	489 ± 203	502 ± 146	205 ± 88	238 ± 84	249 ± 97	353 ± 204	417 ± 233	445 ± 251
Spinal CSF	0 *	0 *	0 *	312 ± 85	300 ± 91	273 ± 90	605 ± 186	581 ± 170	536 ± 205

* Mean spinal CSF flow ~0 over the cardiac cycle.

CSF, cerebrospinal fluid; DV, dual-venc; ICA, internal carotid artery; IJV, internal jugular vein; MV, multi-venc; SV, single-venc; tCBF, total cerebral blood flow; tJBF, total jugular blood flow; VA, vertebral artery; ΔV, volume change.

#### 3.3.1 CSF flow comparisons

**Fig. 7.**
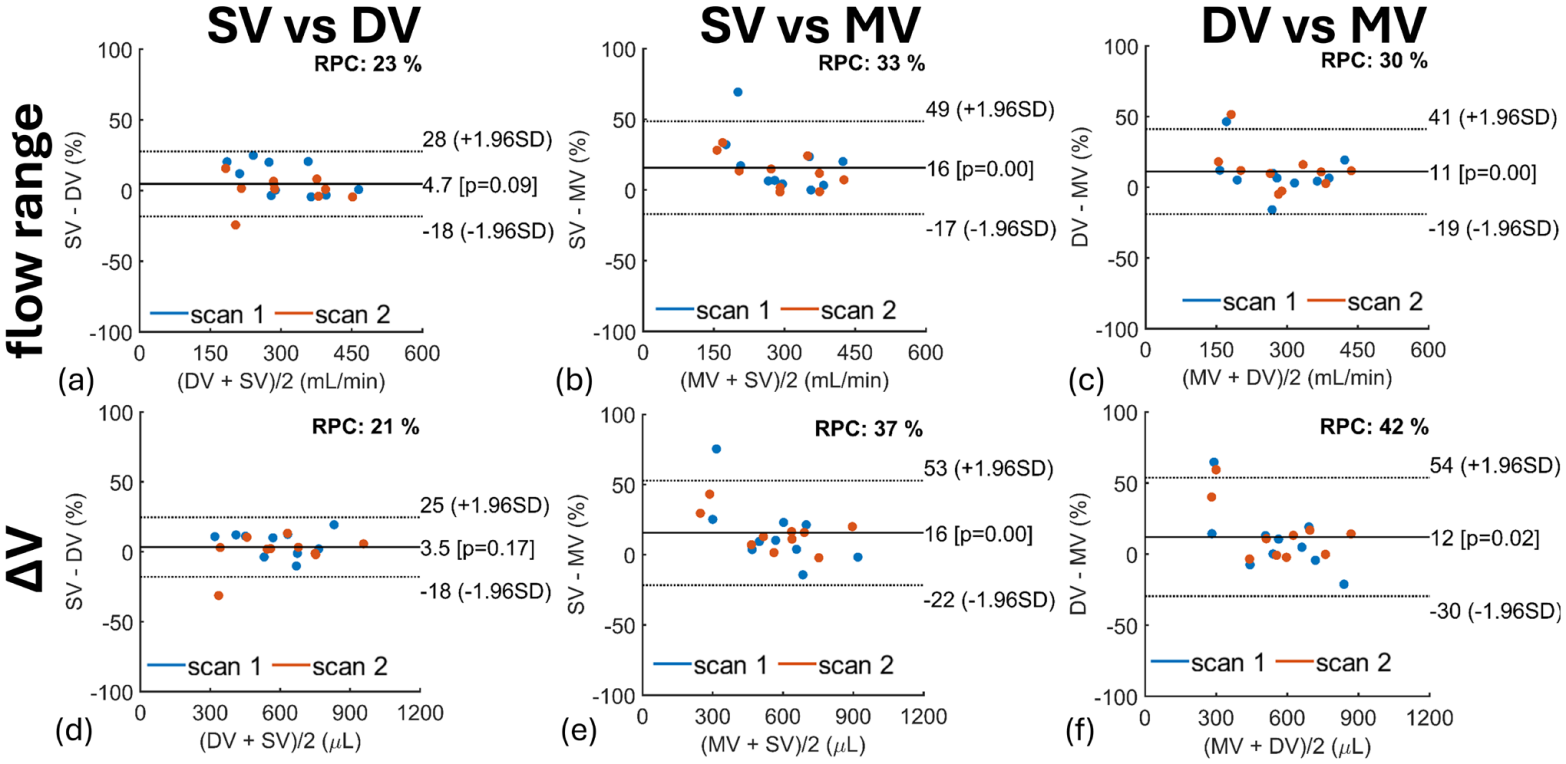
Bland–Altman quantifying the effects of 2D PC flow encoding scheme on spinal canal CSF flow range (top row) and volume change (ΔV) (bottom row) in 10 participants including repeated scans. SC CSF flow range and ΔV were significantly higher in single-venc (SV) (P < 0.001, P < 0.001) and dual-venc (DV) (P < 001, P < 0.001) scans than in multi-venc (MV) scans. Measurement agreement was higher between SV and DV, as indicated by the lower repeatability coefficients (RPC) of 23% ((a) flow range) and 21% ((d) ΔV).

Results from LME models for SC CSF flow range and ΔV are found inTable 2. Flow range and ΔV were statistically lower in multi-venc scans than in single-venc (P < 0.001, P < 0.001) and dual-venc (P < 0.001, P < 0.001) scans, which was also observed in Bland–Altman analyses (Fig. 7). Heart rate significantly influenced ΔV (P < 0.001) (Table 2), with a higher heart rate associated with lower SC CSF ΔV. High intra-class correlation coefficients for flow range (0.94) and ΔV (0.94) indicate variability is primarily across participants and not within participants (repeated scans).

**Table 2.** Linear mixed effects models, intra-class correlation coefficients, and pair-wise comparisons for participant experiments assessing effects of flow encoding scheme and heart rate on spinal canal CSF flow range and volume change (ΔV).

Parameter	Estimate	P-value	Intra-class correlation
SC CSF flow range (mL/min)			0.94
Intercept	312 ± 90 (132, 492)	**<0.001**	-
DV vs. SV	-12 ± 7 (-25, 2)	0.088	-
MV vs. SV	-39 ± 7 (-53, -25)	**<0.001**	-
DV vs. MV	-	**<0.001**	-
HR (bpm)	0 ± 1 (-3, 3)	0.995	-
SC CSF ΔV (µL)			0.94
Intercept	1261 ± 180 (899, 1622)	**<0.001**	-
DV vs. SV	-20 ± 13 (-47, 7)	0.140	-
MV vs. SV	-80 ± 14 (-107, -52)	**<0.001**	-
DV vs. MV	-	**<0.001**	-
HR (bpm)	-10 ± 3 (-15, -5)	**<0.001**	-

Estimate data are ± standard error; data in parentheses are 95% CIs. Bold indicates statistical significance (P < 0.05). Estimate coefficients and CIs from the LME were used for DV versus MV comparisons.

CSF, cerebrospinal fluid; DV, dual-venc; HR, heart rate; MV, multi-venc; LME, linear mixed effects; SC, spinal canal; SV, single-venc; ΔV, volume change.

#### 3.3.2 Blood flow comparisons

**Fig. 8.**
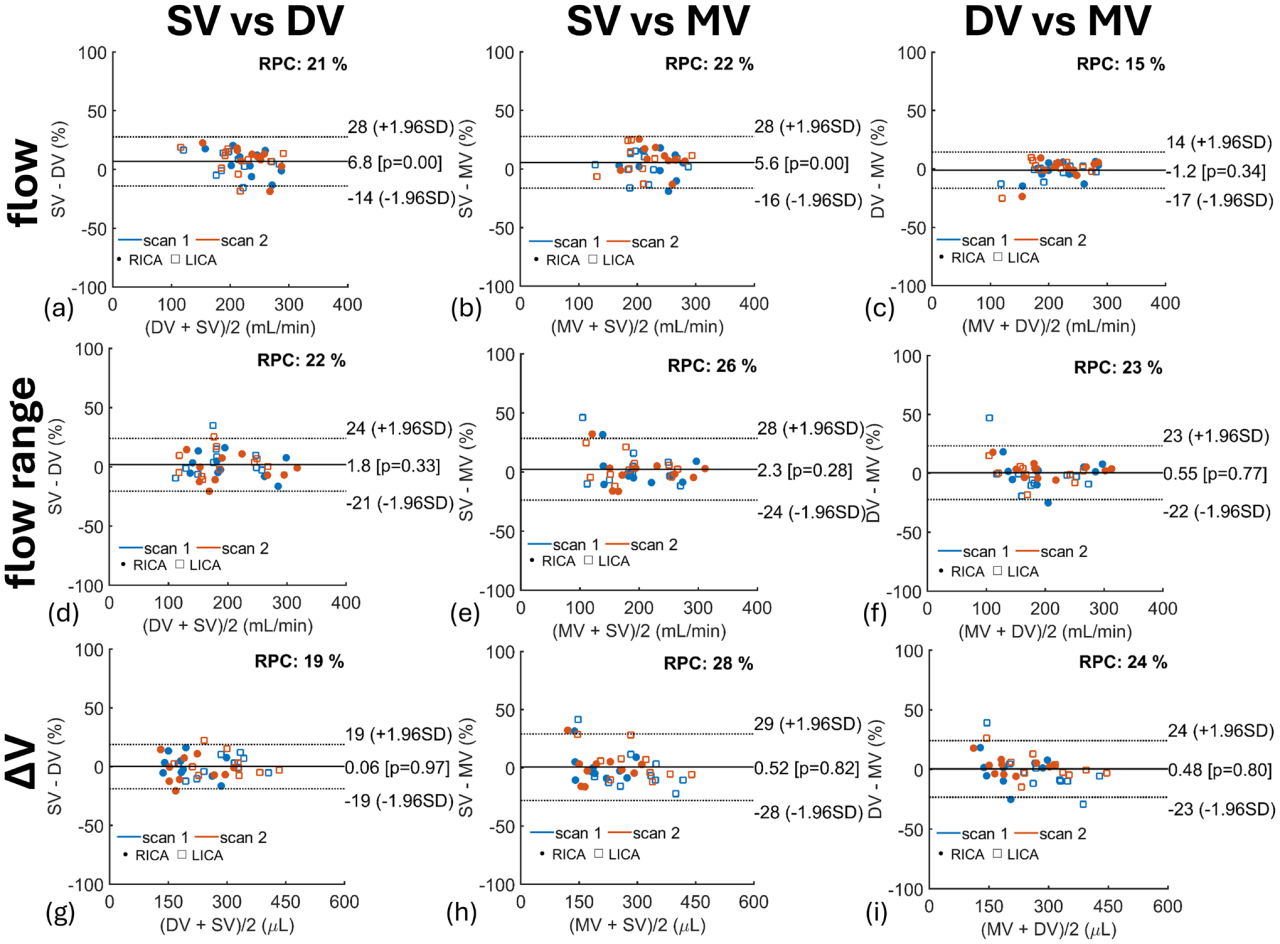
Bland–Altman (BA) quantifying the effects of 2D PC flow encoding scheme on internal carotid arteries (RICA, LICA) blood flow mean (top row), range (middle row), and volume change (ΔV) (bottom row) in 10 participants including repeated scans. Overall, mean flow was significantly higher in single-venc (SV) than in dual-venc (DV) (P < 0.001) and multi-venc (MV) (P < 0.001) scans. Higher repeatability, as indicated by the lower repeatability coefficients (RPC), was measured for mean flow (a, b, c avg. RPC = 19%) than for flow range (d, e, f avg. RPC = 24%) and ΔV (g, h, i avg. RPC = 24%). BA results for VAs and IJVs are summarized inSupplemental Figures 7 and 8.

Results from LME models for ICAs, VAs, and IJVs ΔV are described inTable 3. No significant differences were observed across scan type for tCBF, ICAs, and VAs ΔV. IJVs ΔV was significantly lower in single-venc scans than in dual-venc and multi-venc scans. Heart rate significantly contributed to lower ΔV in both arteries and veins (Table 3). Intra-class correlation coefficients for ΔV measures were higher in arterial blood (≥0.93) than in venous blood (≥0.88). LMEs results for blood flow mean and range can be found inSupplemental Figures 5 and 6. Bland–Altman analyses comparing flow mean, range, and ΔV across scan types for the ICAs are shown inFigure 8. Overall, we observed a significant positive bias for single-venc flow mean (Fig. 8 (a, b, c)) compared with dual-venc and multi-venc scans. No significant bias in ICAs flow range (Fig. 8 (d, e, f)) and ΔV (Fig. 8 (g, h, i)) was measured. Bland–Altman analysis studying differences between blood flow mean, range, and ΔV in the VAs and IJVs can be found inSupplemental Figures 7 and 8.

**Table 3.** Linear mixed effects models, intra-class correlation coefficients, and pair-wise comparisons for participant experiments assessing effects of flow encoding scheme and heart rate on blood flow volume change (ΔV).

Parameter	Estimate	P-value	Intra-class correlation
Total CBF ΔV (µL)			0.95
Intercept	1956 ± 176 (1604, 2308)	**<0.001**	-
DV vs. SV	14 ± 15 (-17, 44)	0.381	-
MV vs. SV	4 ± 16 (-27, 36)	0.789	-
DV vs. MV	-	0.555	-
HR (bpm)	-17 ± 3 (-22, -12)	**<0.001**	-
ICA (R) ΔV (µL)			0.93
Intercept	790 ± 68 (654, 925)	**<0.001**	-
DV vs. SV	7 ± 6 (-5, 20)	0.225	-
MV vs. SV	8 ± 6 (-5, 20)	0.220	-
DV vs. MV	-	0.968	-
HR (bpm)	-7 ± 1 (-9, -5)	**<0.001**	-
ICA (L) ΔV (µL)			0.95
Intercept	652 ± 61 (530, 774)	**<0.001**	-
DV vs. SV	-3 ± 5 (-13, 8)	0.590	-
MV vs. SV	-1 ± 5 (-12, 9)	0.817	-
DV vs. MV	-	0.769	-
HR (bpm)	-6 ± 1 (-7, -4)	**<0.001**	-
VA (R) ΔV (µL)			0.95
Intercept	289 ± 47 (195, 382)	**<0.001**	-
DV vs. SV	4 ± 4 (-4, 12)	0.276	-
MV vs. SV	-3 ± 4 (-11, 5)	0.454	-
DV vs. MV	-	0.073	-
HR (bpm)	-3 ± 1 (-4, -2)	**<0.001**	-
VA (L) ΔV (µL)			0.96
Intercept	228 ± 37 (153, 303)	**<0.001**	-
DV vs. SV	4 ± 3 (-2, 10)	0.208	-
MV vs. SV	-2 ± 3 (-8, 5)	0.596	-
DV vs. MV	-	0.082	-
HR (bpm)	-2 ± 1 (-3, -1)	**0.002**	-
Total JBF ΔV (µL)			0.92
Intercept	1012 ± 196 (619, 1405)	**<0.001**	-
DV vs. SV	64 ± 18 (28, 101)	**<0.001**	-
MV vs. SV	78 ± 19 (41, 115)	**<0.001**	-
DV vs. MV	-	0.454	-
HR (bpm)	-10 ± 3 (-16, -4)	**<0.001**	-
IJV (R) ΔV (µL)			0.88
Intercept	766 ± 156 (452, 1079)	**<0.001**	-
DV vs. SV	40 ± 15 (9, 71)	**0.012**	-
MV vs. SV	66 ± 16 (34, 96)	**<0.001**	-
DV vs. MV	-	0.112	-
HR (bpm)	-8 ± 2 (-13, 3)	**0.001**	-
IJV (L) ΔV (µL)			0.96
Intercept	295 ± 79 (136, 454)	**<0.001**	-
DV vs. SV	29 ± 7 (16, 43)	**<0.001**	-
MV vs. SV	29 ± 7 (15, 42)	**<0.001**	-
DV vs. MV	-	0.553	-
HR (bpm)	-3 ± 1 (-5, 0)	**0.021**	-

Estimate data are ± standard error; data in parentheses are 95% CIs. Bold indicates statistical significance (P < 0.05). Estimate coefficients and CIs from the LME were used for DV versus MV comparisons.

CBF, cerebral blood flow; DV, dual-venc; ICA; internal carotid artery; JBF, jugular blood flow; IJV, internal jugular vein; HR, heart rate; MV, multi-venc; LME, linear mixed effects; SV, single-venc; VA, vertebral artery; ΔV, volume change.

#### 3.3.3 Single-, dual-, and multi-venc scan repeatability and correlations

**Fig. 9.**
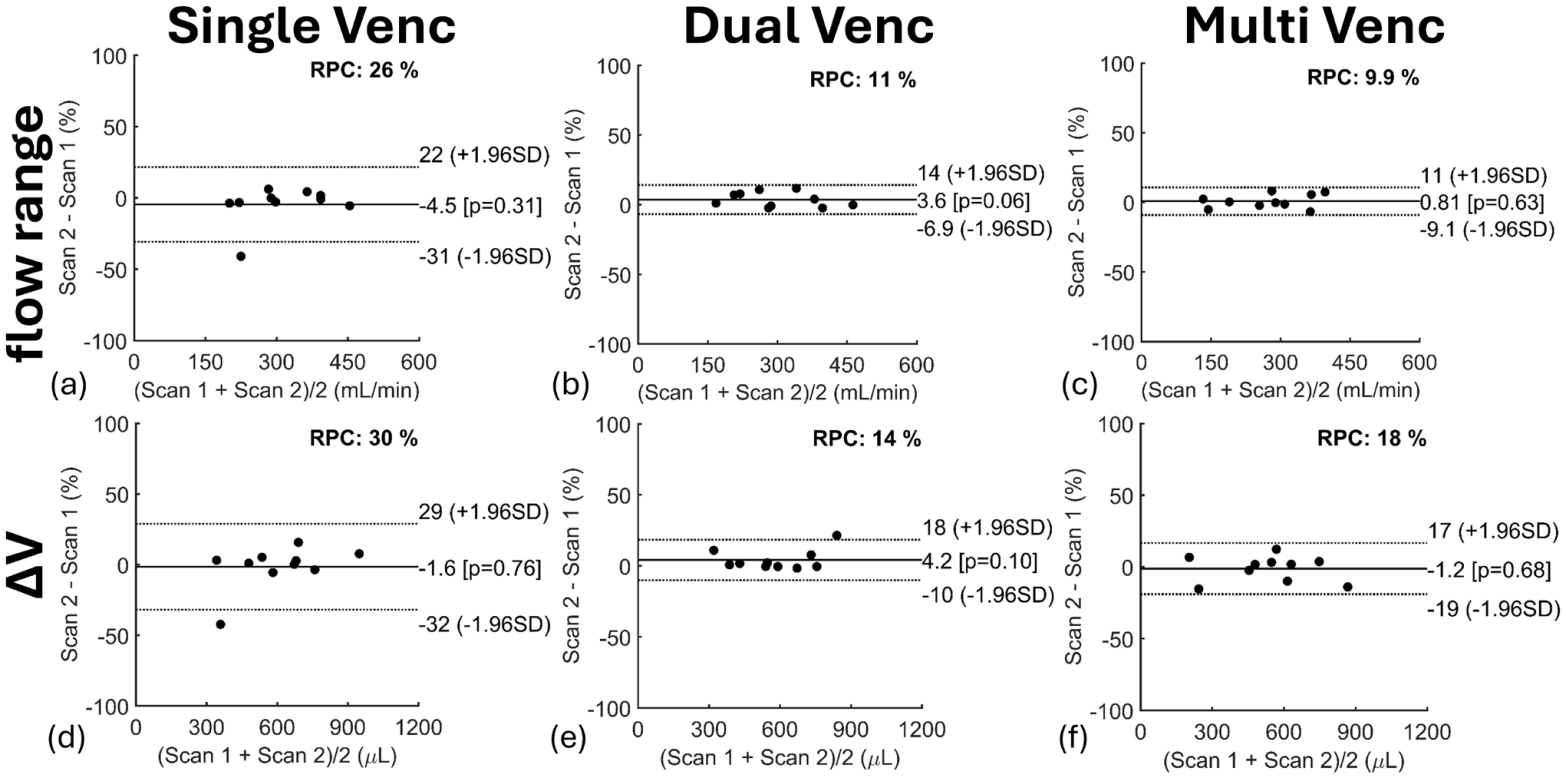
Bland–Altman quantifying repeatability of 2D PC flow encoding schemes on spinal canal CSF flow range (top row) (a, b, c) and pulsatile volume change (ΔV) (bottom row) (d, e, f) in 10 participants. Each scan was repeated twice during the MRI study visit. Overall, no significant bias was observed across repeated measures; however, single-venc (SV) scans showed lower repeatability (higher RPC) than dual-venc (DV) and multi-venc (MV) scans likely from physiological variability as repeated SV scans were not acquired back-to-back compared with DV and MV scans.

**Fig. 10.**
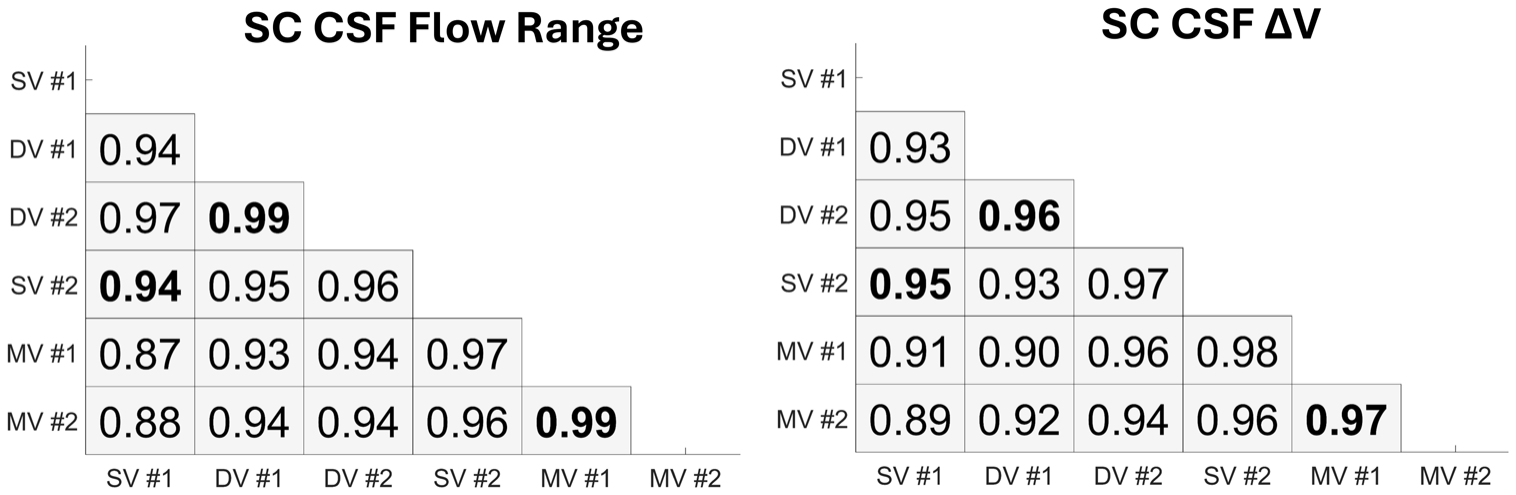
Pearson’s linear correlation coefficients for spinal canal (SC) CSF flow range (left) and pulsatile volume change (ΔV) (right) across flow encoding schemes (single-venc (SV), dual-venc (DV), multi-venc (MV)) and repeated scans. Rows and columns (top-bottom, left right) represent the actual 2D PC scan acquisition order. Repeated scan comparisons are highlighted in bold (e.g., flow range SV #1 vs. SV #2 =**0.94**). Strong correlations were measured across all scans for both flow range (r ≥ 0.87) and ΔV (r ≥ 0.89).

Repeatability of SC CSF flow measures is described inFigures 9and10. Bland–Altman analyses showed higher SC CSF repeatability of flow range and ΔV markers in dual-venc (Fig. 9b, e) (RPC = 11%, RPC = 14%) and multi-venc (Fig. 9c, f) (RPC = 9.9%, RPC = 18%) scans as indicated by their lower RPC values when compared with single-venc scans (Fig. 9a, d) (RPC = 26%, RPC = 30%). Pearson’s correlation coefficients for SC CSF flow range and ΔV (Fig. 10) showed a strong correlation between repeated scans, somewhat stronger for dual-venc (flow range r = 0.99; ΔV r = 0.96) and multi-venc (flow range r = 0.99; ΔV r = 0.97) scans. Correlation across scan types was also strong (flow range r ≥ 0.87; ΔV r ≥ 0.89).

**Fig. 11.**
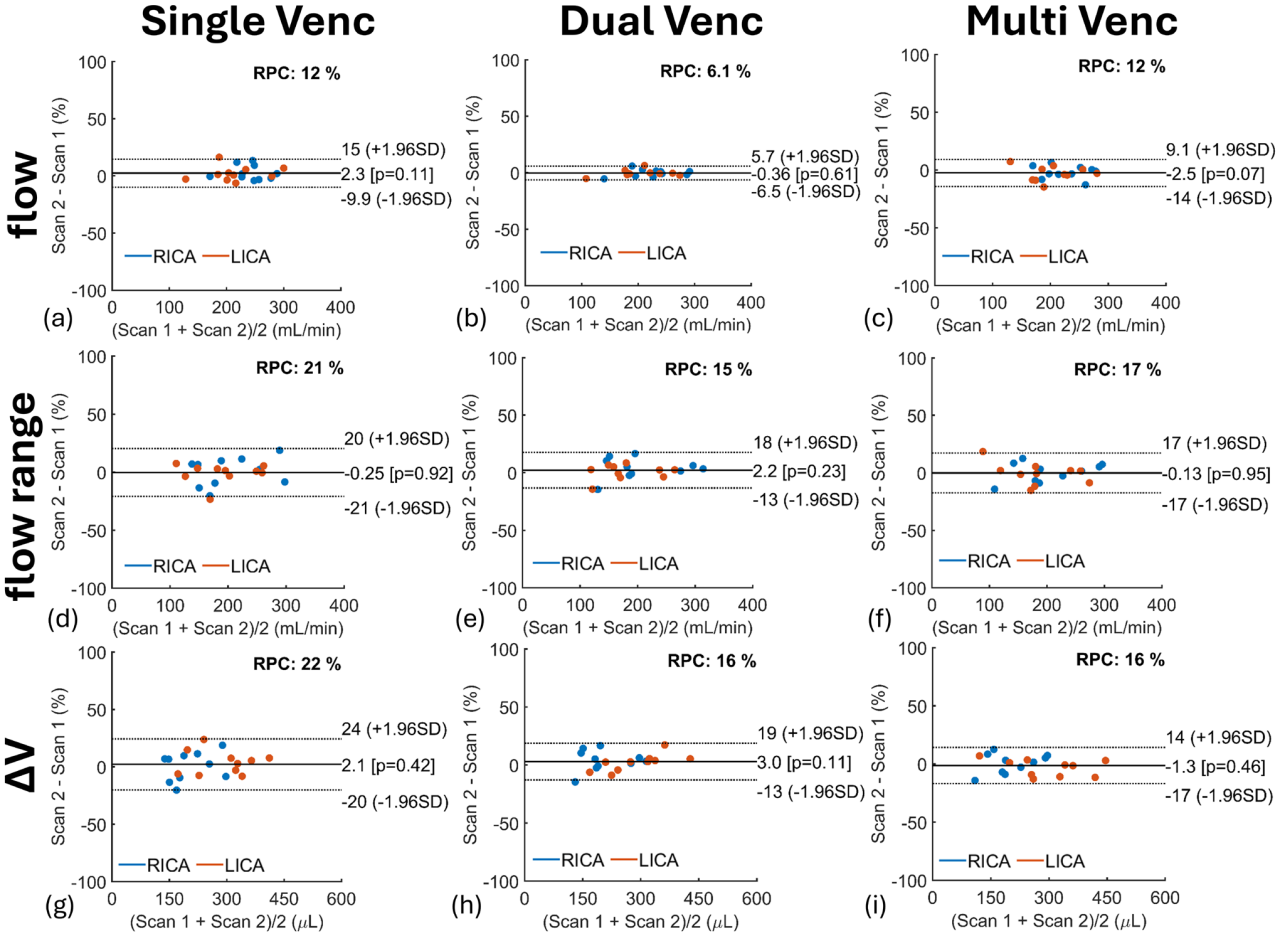
Bland–Altman (BA) quantifying repeatability of 2D PC flow encoding schemes on internal carotid arteries (RICA, LICA) blood flow mean (top row) (a, b, c), range (middle row) (d, e, f), and volume change (ΔV) (bottom row) (g, h, i) in 10 participants. Each scan was repeated twice during the MRI study visit. Overall, no significant bias was observed across repeated measures; however, single-venc (SV) scans showed lower repeatability than dual-venc (DV) and multi-venc (MV) scans in cardiac-resolved measures such as flow range and ΔV likely from physiological variability as repeated SV scans were not acquired back-to-back. BA repeatability results for vertebral arteries and internal jugular veins are summarized inSupplemental Figures 9 and 10.

Bland–Altman analyses assessing repeatability of ICAs blood flow mean, range, and ΔV from repeated scans are shown inFigure 11. The blood flow mean marker showed the highest repeatability for all flow encoding schemes: single-venc (Fig. 11a) (RPC = 12%), dual-venc (Fig. 11b) (RPC = 6.1%), and multi-venc (Fig. 11c) (RPC = 12%) scans. Bland–Altman repeatability results for VAs and IJVs are summarized inSupplemental Figures 9 and 10. Pearson’s correlation coefficients for ICAs, VAs, and IJVs blood flow markers are shown inFigure 12. Repeated scans correlations were highest for dual-venc and multi-venc scans, for example, dual-venc arterial flow mean (r = 0.99), range (r ≥ 0.98), and ΔV (r ≥ 0.98), and venous flow mean (r = 1.00), range (r = 0.98), and ΔV (r = 0.98). Strong correlations were measured across all scans for arterial flow mean (r ≥ 0.83), range (r ≥ 0.91), and ΔV (r ≥ 0.94) and venous flow mean (r ≥ 0.92), range (r ≥ 0.91), and ΔV (r ≥ 0.91).

**Fig. 12.**
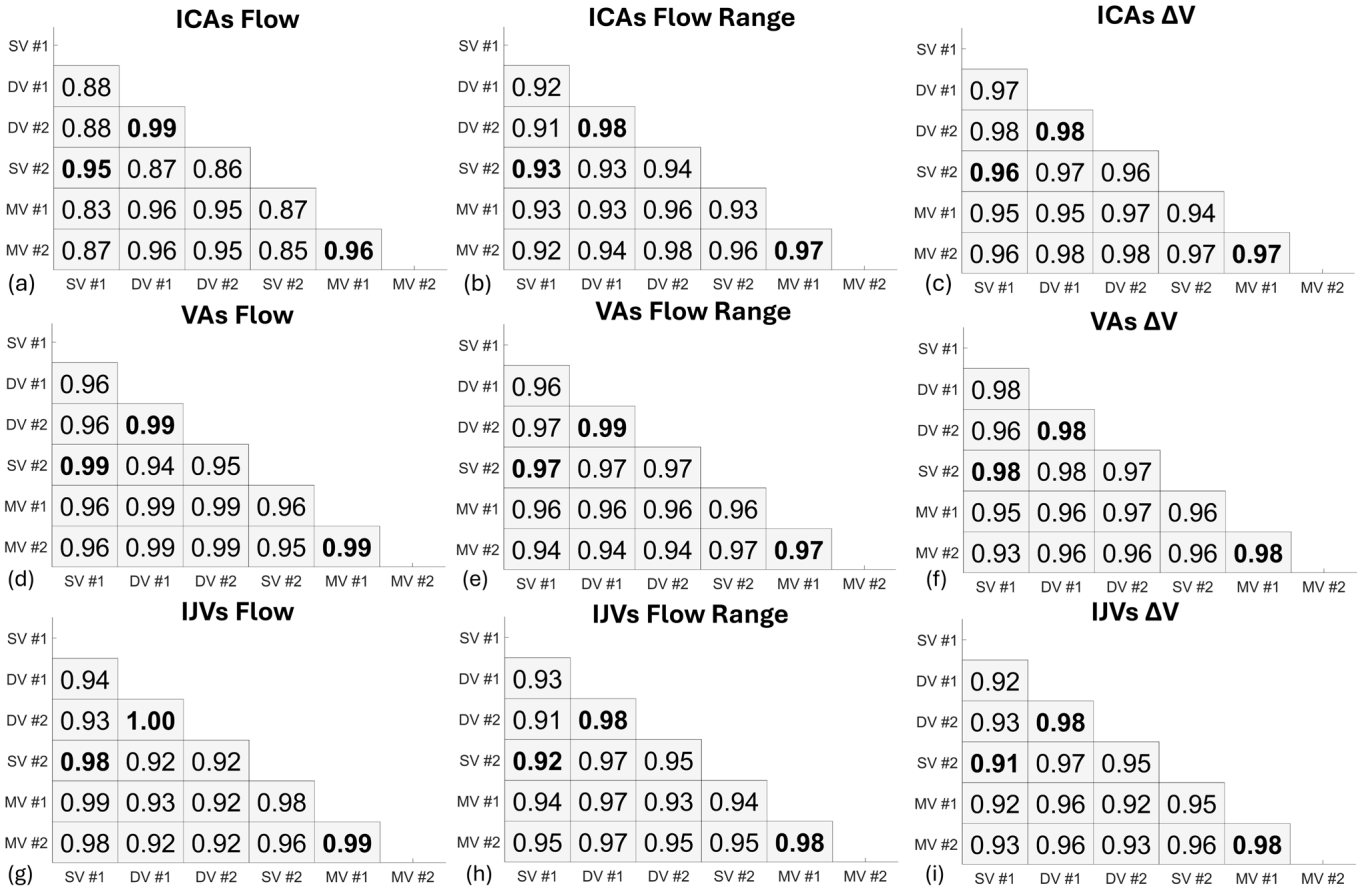
Pearson’s linear correlation coefficients for internal carotid arteries (ICAs) (top row), vertebral arteries (VAs) (middle row), and internal jugular veins (IJVs) (bottom row) blood flow mean (left column), range (middle column), and volume change (ΔV) (right column) across flow encoding schemes (single-venc (SV), dual-venc (DV), multi-venc (MV)), and repeated scans. For each subplot, the order of rows and columns (top-bottom, left right) represents the scanning order. Bold numbers highlight repeated scan comparisons (e.g., ICAs Flow SV#1 vs. SV #2 =**0.95**). Strong correlations were measured across all scans for arterial flow mean (r ≥ 0.83), range (r ≥ 0.91), and ΔV (r ≥ 0.94) and venous flow mean (r ≥ 0.92), range (r ≥ 0.91), and ΔV (r ≥ 0.91). Repeated scans correlations were highest with DV arterial flow mean (r = 0.99), range (r ≥ 0.98), and ΔV (r ≥ 0.98), and venous flow mean (r = 1.00), range (r = 0.98), and ΔV (r = 0.98).

## 4 Discussion

In this study, we proposed and investigated an interleaved multiple venc flow encoding 2D PC MRI approach for the synchronous characterization of neurofluid flow. We conducted comparison studies against standard asynchronous 2D PC imaging in 10 participants. The results demonstrated good overall agreement between the standard asynchronous single-venc scans and the proposed interleaved dual-venc and multi-venc scans, with high repeatability. Notably, quantification of the net blood flow and CSF flow pulsatile volume change relationship from interleaved dual-venc and multi-venc scans showed similar and stronger coupling associations than results from asynchronous single-venc scans. A consistently longer lag between SC-VAs CSF and blood flow, compared with the SC-ICAs lag, was observed at the level of the C1/C2 vertebrae across all three scan types (single-, dual-, and multi-venc). The multi-venc scans appeared to underestimate the SC CSF flow range and ΔV compared with single- and dual-venc scans, likely due to a higher degree of undersampling and blood flow-favorable flip angle in the multi-venc protocol. Overall, the study supports that 2D PC MRI from synchronous dual-venc and multi-venc scans can perform similarly to single-venc scans and may offer improved depiction of cardiac-resolved neurofluid dynamics over asynchronous single-venc scans.

Linear regression models studying the relationship between net blood flow (tCBF – tJBF) and CSF flow (SC) pulsatile volume change ΔV showed similar coefficient of determination for single-venc (R^2^= 0.71) and dual-venc scans (R^2^= 0.70). These results indicate good agreement between the proposed methods and the standard single-venc approach. The somewhat higher coefficient of determination measured in multi-venc scans (R^2^= 0.78) indicates multi-venc scans might benefit from higher VNR and phase unwrapping from the middle-venc (50 cm/s) and high-venc (100 cm/s) flow encodes. In this study, in all scans there was a positive difference between net blood flow and CSF flow ΔV, which might indicate incomplete capture of venous flows or underestimation of CSF flow. Previous studies have shown there can be significant venous drainage (e.g., 10–30%) through smaller veins such as the epidural, vertebral, and deep cervical veins (Pomschar et al., 2013), which might explain our observations. Furthermore, single directional velocity encoding could be challenged by IJV tortuosity limiting orthogonal plane prescription to the flows of interest. In addition, CSF flow quantification might have been influenced by the utilization of a blood flow favorable flip angle (8°) across all the protocols.

Cross-correlation was used to study cardiac coupling between VAs and ICAs blood flow and SC CSF flow. A significantly longer lag was measured for SC-VAs than for SC-ICAs across all scans including single-venc, dual-venc, and multi-venc, with lags of ~19 ms for SC-VAs and ~5 ms for SC-ICAs. Similar observations have been reported previously in other 2D PC MRI studies, where VAs flow preceded ICAs flow at the cranio-spinal level (Balédent et al., 2004;Ford et al., 2005). Previous studies have estimated peak SC CSF flow occurred at 5% of the cardiac cycle after ICA peak flow (Balédent et al., 2004), while our study indicates a much shorter lag between SC CSF flow and ICAs blood flow of ~0.5% of the cardiac cycle. The measured shorter SC-ICAs lag in this study is likely attributed to the use of higher temporal resolution (30 vs. 16 cardiac phases) and another lag detection algorithm. Here we used a cross-correlation approach compared with a peak detection approach to estimate lags. Peak detection approaches have been shown to be susceptible to noise and nonphysiological changes compared with cross-correlation which uses the entire waveform to estimate lags (Wentland et al., 2013).

Quantitative evaluation of arterial, venous, and CSF flow measurements agrees well with previous studies (Liu et al., 2022,^2024^;Wåhlin et al., 2012). Statistical models showed significant effects from heart rate which are consistent with previous studies investigating the influence of heart rate on macroscopic blood and CSF flow at the cervical level (Daouk et al., 2017). Higher heart rates were associated with lower ΔV for blood and CSF, which may indicate a lower stroke volume from less time for the heart chambers to fill with blood between beats (Fletcher et al., 1995). SC CSF flow range and ΔV were statistically lower in multi-venc scans than in single-venc and dual-venc scans. During the dual-venc and multi-venc protocol design, a blood flow favorable flip angle of 8° was heuristically selected after considering various factors such as inflow effects and the size of areas of interest (e.g., ICAs vs. SC area). Multi-venc scans CSF measures may have been challenged by the combined effect from higher undersampling and selection of flip angle. Multi-venc scans required a higher number of flow encodes than the single- and dual-venc scans. To maintain a similar scan time of 1 min, less measurements per flow encode were collected for multi-venc scans (e.g., 1500 spiral arms compared with 2000 (dual-venc) and 3300 (single-venc)). Not accounting for g-factor effects and with SNR ~ √N, where N is the number of samples, natively the multi-venc scans SNR was 33% and 13% lower than single- and dual-venc scans, respectively. In addition to the lower SNR in multi-venc scans, from the SPGR signal model, we can estimate the effect of the flip angle selection on the CSF signals. This results in M_xy CSF_~ 1.28 M_xy Study_, indicating a 28% higher CSF signal in multi-venc scans if we had used Ernst angle for T1 of CSF (4°), instead of the study flip angle of 8°. However, a lower study flip angle would have had a negative impact on blood flow measurements. Future studies may consider strategies to optimize flip angle based on flow encode, while ensuring consistent signal across the imaging slice. In our study, we did not report the mean flow values of SC CSF over the cardiac cycle. Although the values were small and approximate to 0, we could not rule out the possibility that the measurements merely reflected residual background offset error and small respiratory effects (Spijkerman et al., 2019).

A flow offset was measured between single-venc and the dual-venc and multi-venc scans. The offset can be observed more pronounced in the venous flow profiles depicted inFigure 3. We attributed this offset to incomplete removal of background phase presumably from uncompensated eddy currents in the single-venc scans, which can be observed inSupplemental Figure 2velocity images. In our approach, dual-venc and multi-venc scans used one-sided flow encoding to reduce the number of encodes, while single-venc scans used two-side (e.g., +, -) encoding to achieve the shortest possible TEs and TRs (Pelc et al., 1991). In the one-sided encoding scheme used in dual- and multi-venc scans, a flow compensated encode (shared reference measurement) was acquired followed by flow encodes with bipolar gradients that achieve the desirable vencs. In the two-sided encoding scheme used in single-venc scans, a pair of encodes with smaller bipolar gradients were played with opposite polarities and combined to achieve the prescribed venc. This approach enables shorter TEs and TRs due to the smaller first gradient moments requirement; however, it lacks a flow compensated encode measurement which can lead to lower quality of the magnitude and angiographic images, and lower effective temporal resolution (Pelc et al., 1991). Offsets were larger in veins which have slower velocities relative to venc, increasing sensitivity to residual background phase offsets. In our study, we fit a polynomial to the phase of stationary tissue to remove the background phase. However, a challenge of neck compared with brain neurofluid imaging is the limited amount of stationary tissue available to fit this phase (Busch et al., 2017;Minderhoud et al., 2020). Alternatively, approaches leveraging machine learning to remove background phase could be explored (Kim et al., 2023;Srinivas et al., 2023). More recently, field cameras have been utilized to measure systems gradient impulse response function to compensate for background phase offsets (Loecher & Ennis, 2025).

Bland–Altman analyses and Pearson correlations indicate high repeatability of 2D PC MRI (Morgan et al., 2023). In our study, repeatability and correlation coefficients were higher for dual-venc and multi-venc scans than for single-venc scans, likely due to the scan acquisition order. While dual-venc and multi-venc scan repeats were acquired back-to-back, single-venc repeats were not, introducing potential variability from heart rate and physiology. Single-venc scanning order prioritized collecting the 75 cm/s and 8 cm/s scans sequentially, rather than back-to-back repeated scans, to facilitate a more similar ensemble of heart beats between blood and CSF flow. The average heart rate difference between repeated dual-venc scans was ~0.2 bpm, compared with ~2 bpm between repeated single-venc (75 cm/s) scans (Supplemental Fig. 1). High intra-class correlation coefficients suggest measures variance was dominated by differences across participants rather than between repeated scans, also indicative of high measurement repeatability. Venous measurements generally displayed lower intra-class correlations and reproducibility compared with arterial and CSF results. This observation in veins may relate to their lower pulsatility, noisier measurement nature from lower velocities relative to venc, and higher sensitivity to respiratory-induced pressure effects (Owashi et al., 2024).

In this study, we employed a multi-venc scan protocol which offers the opportunity to unwrap velocities in the middle-venc encode using the high-venc encode, while preserving potential gains in VNR (Johnson & Markl, 2010). Multi-venc scans are also likely valuable in scenarios where a wider velocity dynamic range is required (e.g., very slow CSF flow). For example, the lateral ventricles typically display very slow CSF flow velocities compared with spinal canal and cerebral aqueduct (Vikner et al., 2024). In such scenarios, a multi-venc protocol optimized for both blood and wide velocity range of CSF signals might be useful. To probe cranio-spinal blood and CSF flow dynamics, dual-venc scans might be beneficial and sufficient. While dual-venc scans have typically been used for blood flow imaging, the proposed dual-venc scans incorporate interleaved encoding of blood and CSF flows. This approach enables synchronous physiological measurements across neurofluids and differs from traditional dual-venc acquisitions, which essentially collect back-to-back single-venc scans (Schnell et al., 2017;Tang et al., 2024).

This study and proposed method have several limitations. Dual-venc and multi-venc protocols require simultaneous imaging of blood and CSF, and thus the flip angle must be identical for both flows. This leads to a compromised selection of flip angle, balancing the higher optimal flip angle for blood and low optimal flip angle for CSF. Studies investigating improved optimization of such imaging parameters and acquisition schemes are warranted. Further studies are needed to investigate physiological variations. For example, single-venc scans were not acquired in a back-to-back fashion to reduce the variability between single-venc high- and low-venc scans. This may have led to greater physiological variability between repeated single-venc scans. Despite similar heart rates, respiratory, blood pressure, and other system physiological effects were not considered in this study.

## 5 Conclusion

In conclusion, this work investigates a 2D PC MRI approach for the synchronous measurement of cranio-spinal arterial, venous, and CSF flows. Interleaved dual-venc and multi-venc scans demonstrated high measurement repeatability and strong cardiac coupling between neurofluids when compared with asynchronous single-venc scans. In participant experiments, a consistently shorter SC-ICAs lag (~5 ms) was measured, compared with the significantly longer SC-VAs lag (~19 ms) across all scan types (single-, dual-, and multi-venc). Overall, the study supports that interleaved dual- and multi-venc 2D PC MRI might enable improved investigation of cardiac-driven neurofluid dynamics over asynchronous single-venc approaches.

## Supplementary Material

Supplementary Material

## Data Availability

The datasets used and/or analyzed during the current study are available from the corresponding author on reasonable request.
