# Supplementary material for: Simultaneous and synchronous characterization of blood and CSF flow dynamics using multiple Venc PC MRI
Source: Imaging Neurosci (Camb). 2025 Mar 27;3:imag_a_00521. doi: 10.1162/imag_a_00521 (PMC12319981; doi:10.1162/imag_a_00521)
Supplement: Supplementary Material [file imag_a_00521-supp.pdf]

Supplemental Tables and Figures

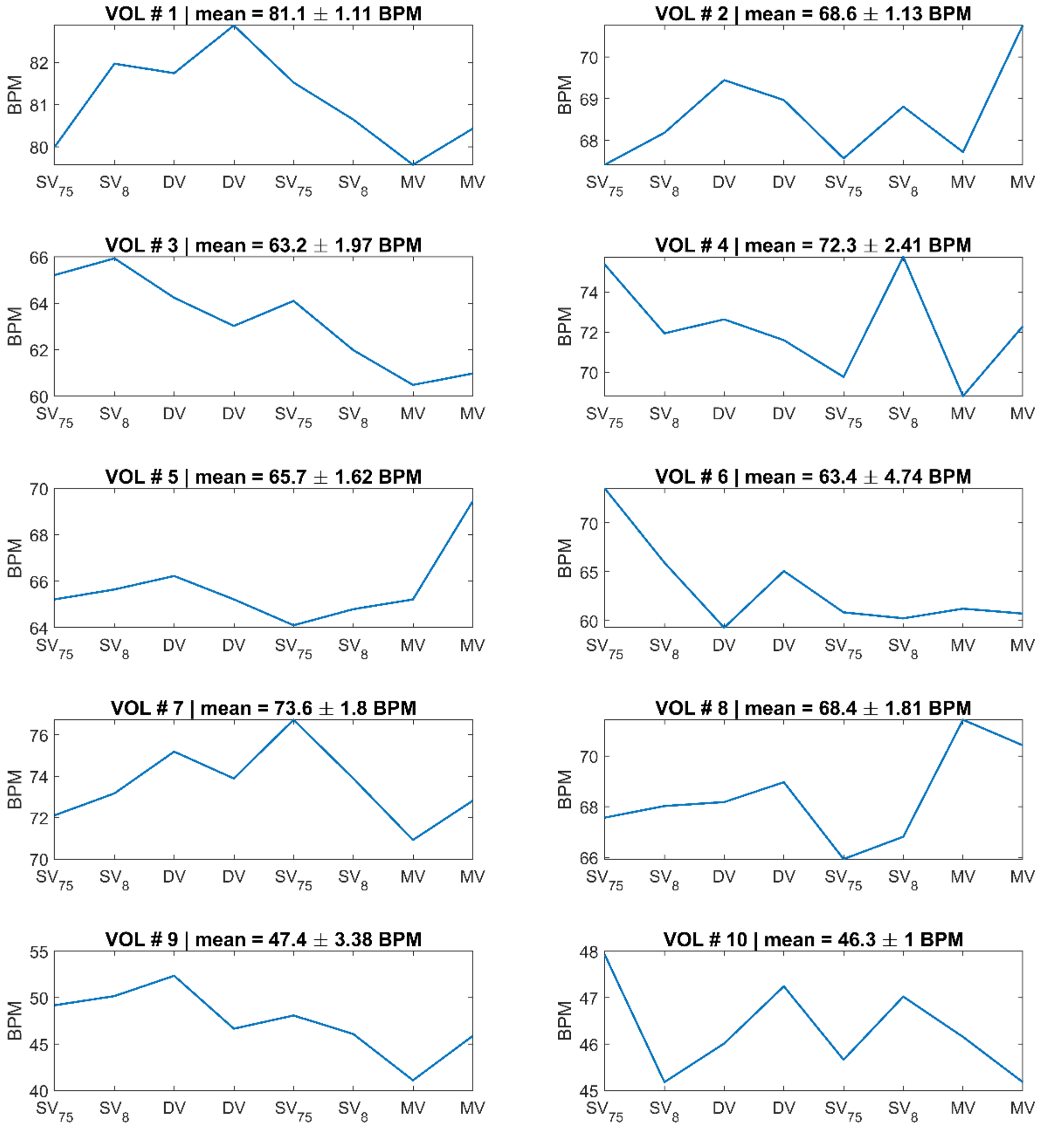

Supplemental Figure 1: Heart rate (HR) measurements from photoplethysmography for each scan for every participant summarizing HR across the 2D Phase Contrast MRI protocol.

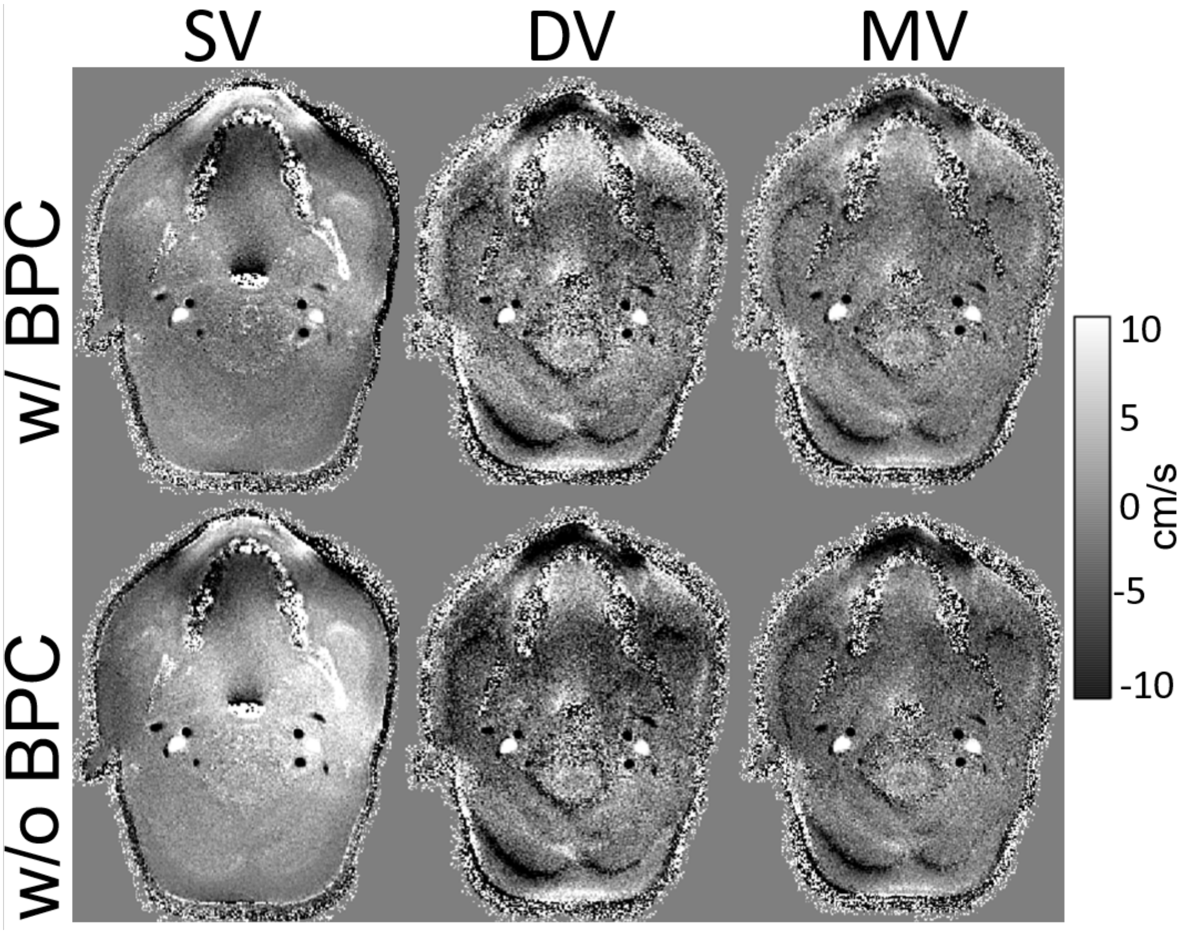

Supplemental Figure 2: Example of velocity images from a participant for single venc (SV, venc = 75 cm/s), dual venc (DV, venc = 75 cm/s) and multi venc (MV, venc = 50 cm/s) encoding schemes. Images are shown with (top) and without (bottom) second order polynomial background phase correction (BPC). While BPC removed substantial phase offset (top vs bottom), residual uncorrected phase offset (top) were noticeable across all images and different between SV and the DV and MV scans. These residual phase offsets are likely major contributors to mean flow differences between SV, and the interleaved DV and MV scans. Complete BPC of neck scans is challenging because of the limited availability of static tissue to fit the background phase, which is less of a concern in 2D PC brain imaging.

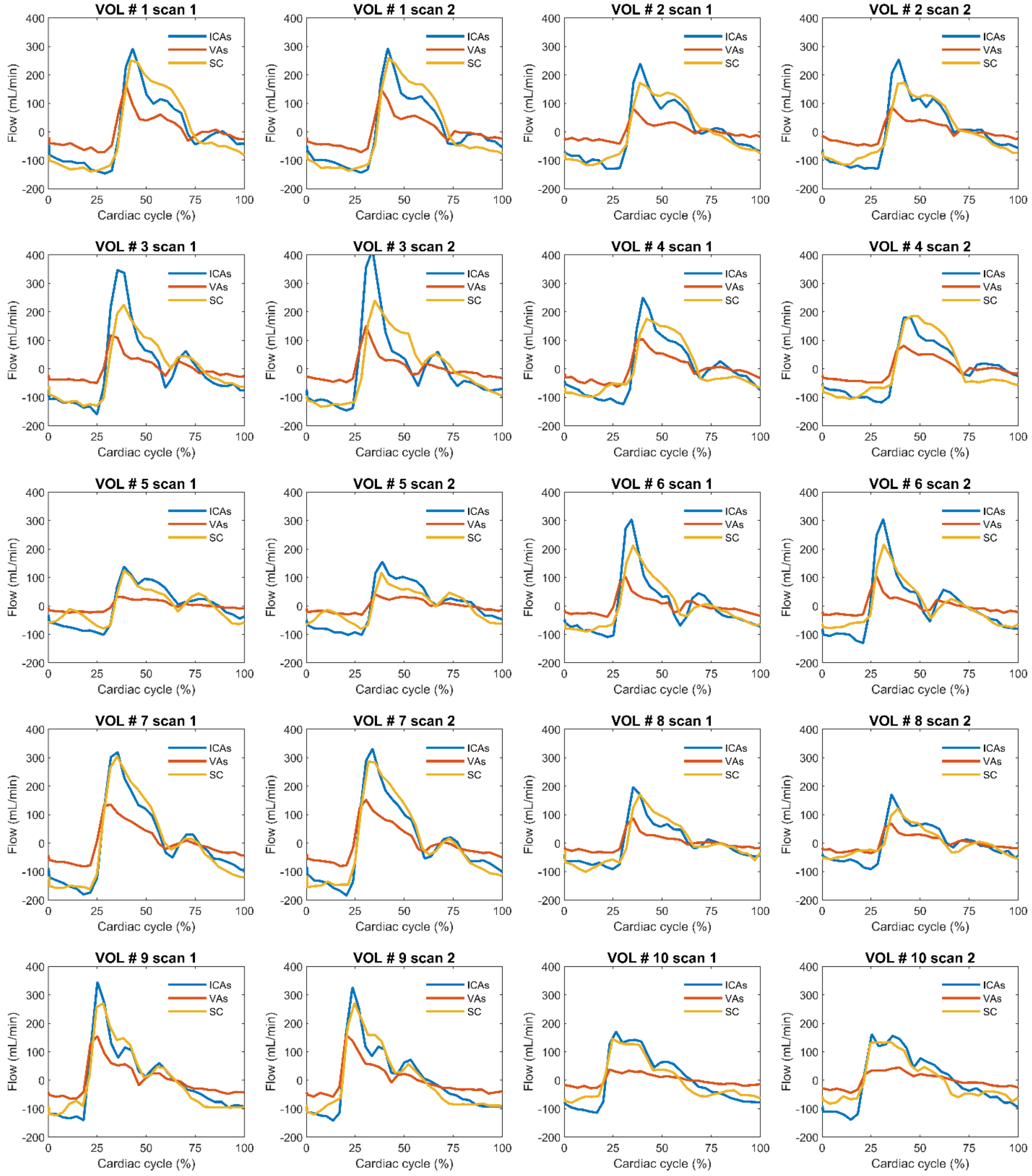

Supplemental Figure 3: Single venc (SV) demeaned cardiac-resolved internal carotid arteries (ICAs) and vertebral arteries (VAs) blood flow and spinal canal (SC) CSF flow profiles for all 10 participants and repeated scans used to study the temporal lag between ICAs, VAs inflow and SC flow.

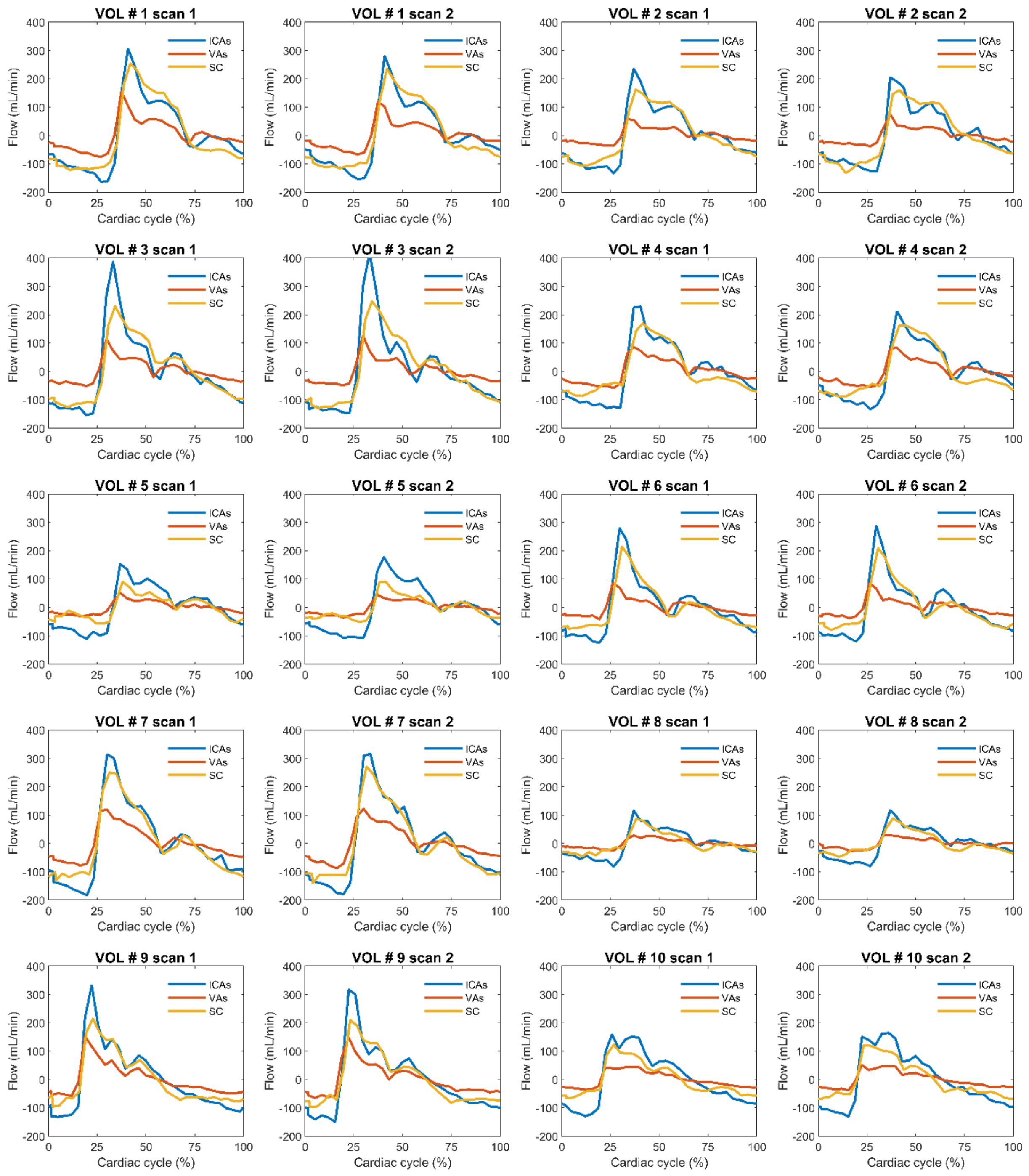

Supplemental Figure 4: Multi venc (MV) demeaned cardiac-resolved internal carotid arteries (ICAs) and vertebral arteries (VAs) blood flow and spinal canal (SC) CSF flow profiles for all 10 participants and repeated scans used to study the temporal lag between ICAs inflow and SC outflow. Similar waveform shapes were observed across flow encoding schemes.

Supplemental Figure 5: Linear mixed effects models, intra-class correlation coefficients, and pair-wise comparisons for participant experiments assessing effects of flow encoding scheme and heart rate on blood flow mean.

Parameter	Estimate	P-value	Intra-class correlation
Total CBF (mL/min)			0.93
intercept	845 ± 113 (618, 1073)	**<0.001**	-
DV vs SV	-60 ± 10 (-81, -40)	**<0.001**	-
MV vs SV	-58 ± 10 (-79, -37)	**<0.001**	-
DV vs MV	-	0.929	-
HR (bpm)	-3 ± 2 (-6, 0)	0.055	-
ICA (R) Blood Flow (mL/min)			0.88
intercept	342 ± 43 (256, 428)	**<0.001**	-
DV vs SV	-16 ± 4 (-24, -7)	**<0.001**	-
MV vs SV	-16 ± 4 (-25, -7)	**<0.001**	-
DV vs MV	-	0.929	-
HR (bpm)	-2 ± 1 (-3, 0)	**0.018**	-
ICA (L) Blood Flow (mL/min)			0.95
intercept	291 ± 41 (208, 374)	**<0.001**	-
DV vs SV	-11 ± 4 (-18, -4)	**0.003**	-
MV vs SV	-12 ± 4 (-19, -5)	**0.002**	-
DV vs MV	-	0.804	-
HR (bpm)	-1 ± 1 (-2, 0)	0.051	-
VA (R) Blood Flow (mL/min)			0.95
intercept	122 ± 19 (82, 161)	**<0.001**	-
DV vs SV	-16 ± 2 (-20, -13)	**<0.001**	-
MV vs SV	-16 ± 2 (-22, -13)	**<0.001**	-
DV vs MV	-	0.935	-
HR (bpm)	-1 ± 0 (-1, 0)	0.051	-
VA (L) Blood Flow (mL/min)			0.97
intercept	107 ± 29 (50, 164)	**<0.001**	-
DV vs SV	-17 ± 2 (-22, -12)	**<0.001**	-
MV vs SV	-14 ± 2 (-19, -9)	**<0.001**	-
DV vs MV	-	0.209	-
HR (bpm)	0 ± 0 (-1, 1)	0.633	-
Total JBF (mL/min)			0.93
intercept	364 ± 153 (58, 671)	**0.021**	-
DV vs SV	87 ± 14 (59, 115)	**<0.001**	-
MV vs SV	101 ± 14 (73, 129)	**<0.001**	-
DV vs MV	-	0.325	-
HR (bpm)	1 ± 2 (-4, 5)	0.797	-
IJV (R) Blood Flow (mL/min)			0.89
intercept	231 ± 126 (-21, 484)	0.072	-
DV vs SV	48 ± 12 (23, 73)	**<0.001**	-
MV vs SV	65 ± 13 (39, 90)	**<0.001**	-
DV vs MV	-	0.185	-
HR (bpm)	0 ± 2 (-3, 4)	0.819	-
IJV (L) Blood Flow (mL/min)			0.97
intercept	152 ± 68 (15, 289)	**0.031**	-
DV vs SV	39 ± 6 (28, 50)	**<0.001**	-
MV vs SV	36 ± 6 (25, 47)	**<0.001**	-
DV vs MV	-	0.576	-
HR (bpm)	0 ± 1 (-2, 2)	0.880	-

Estimate data are ± standard error; data in parentheses are 95% CIs. Abbreviations: CBF, cerebral blood flow; DV, dual venc; ICA; internal carotid artery; JBF, jugular blood flow; IJV, internal jugular vein; HR, heart rate; MV, multi venc; LME, linear mixed effects; SV, single venc; VA, vertebral artery. Bold indicates statistical significance ($P<0.05$). Estimate coefficients and CIs from the LME were used for DV vs MV comparisons.

Supplemental Figure 6: Linear mixed effects models, intra-class correlation coefficients, and pair-wise comparisons for participant experiments assessing effects of encoding scheme and heart rate on blood flow range.

Parameter	Estimate	P-value	Intra-class correlation
Total CBF Range (mL/min)			0.95
intercept	478 ± 114 (248, 708)	**<0.001**	-
DV vs SV	-11 ± 10 (-30, 9)	0.283	-
MV vs SV	-14 ± 10 (-34, 7)	0.184	-
DV vs MV	-	0.777	-
HR (bpm)	1 ± 2 (-3, 4)	0.684	-
ICA (R) Blood Flow Range (mL/min)			0.94
intercept	221 ± 52 (116, 325)	**<0.001**	-
DV vs SV	3 ± 5 (-6, 12)	0.519	-
MV vs SV	0 ± 5 (-9, 9)	0.997	-
DV vs MV	-	0.532	-
HR (bpm)	0 ± 1 (-2, 1)	0.727	-
ICA (L) Blood Flow Range (mL/min)			0.94
intercept	167 ± 45 (75, 259)	**<0.001**	-
DV vs SV	-9 ± 4 (-17, -1)	**0.031**	-
MV vs SV	-5 ± 4 (-13, 3)	0.244	-
DV vs MV	-	0.332	-
HR (bpm)	0 ± 1 (-1, 2)	0.596	-
VA (R) Blood Flow Range (mL/min)			0.95
intercept	93 ± 29 (35, 151)	**0.002**	-
DV vs SV	-2 ± 3 (-7, 3)	0.493	-
MV vs SV	-7 ± 3 (-13, -3)	**0.005**	-
DV vs MV	-	**0.031**	-
HR (bpm)	0 ± 0 (-1, 0)	0.398	-
VA (L) Blood Flow Range (mL/min)			0.95
intercept	30 ± 32 (-33, 94)	0.347	-
DV vs SV	-1 ± 3 (-6, 5)	0.744	-
MV vs SV	-5 ± 3 (-11, 0)	0.056	-
DV vs MV	-	0.109	-
HR (bpm)	1 ± 0 (0, 2)	0.073	-
Total JBF Range (mL/min)			0.90
intercept	310 ± 89 (131, 488)	**<0.001**	-
DV vs SV	33 ± 9 (16, 50)	**<0.001**	-
MV vs SV	41 ± 9 (24, 58)	**<0.001**	-
DV vs MV	-	0.363	-
HR (bpm)	-2 ± 1 (-4, 1)	0.225	-
IJV (R) Blood Flow Range (mL/min)			0.91
intercept	253 ± 79 (94, 413)	**0.002**	-
DV vs SV	27 ± 8 (12, 42)	**<0.001**	-
MV vs SV	32 ± 8 (16, 47)	**<0.001**	-
DV vs MV	-	0.509	-
HR (bpm)	-2 ± 1 (-4, 1)	0.171	-
IJV (L) Blood Flow Range (mL/min)			0.93
intercept	83 ± 42 (-2, 167)	0.056	-
DV vs SV	17 ± 4 (9, 24)	**<0.001**	-
MV vs SV	17 ± 4 (9, 25)	**<0.001**	-
DV vs MV	-	0.951	-
HR (bpm)	0 ± 1 (-1, 1)	0.739	-

Estimate data are ± standard error; data in parentheses are 95% CIs. Abbreviations: CBF, cerebral blood flow; DV, dual venc; ICA; internal carotid artery; JBF, jugular blood flow; IJV, internal jugular vein; HR, heart rate; MV, multi venc; LME, linear mixed effects; SV, single venc; VA, vertebral artery. Bold indicates statistical significance ($P<0.05$). Estimate coefficients and CIs from the LME were used for DV vs MV comparisons.

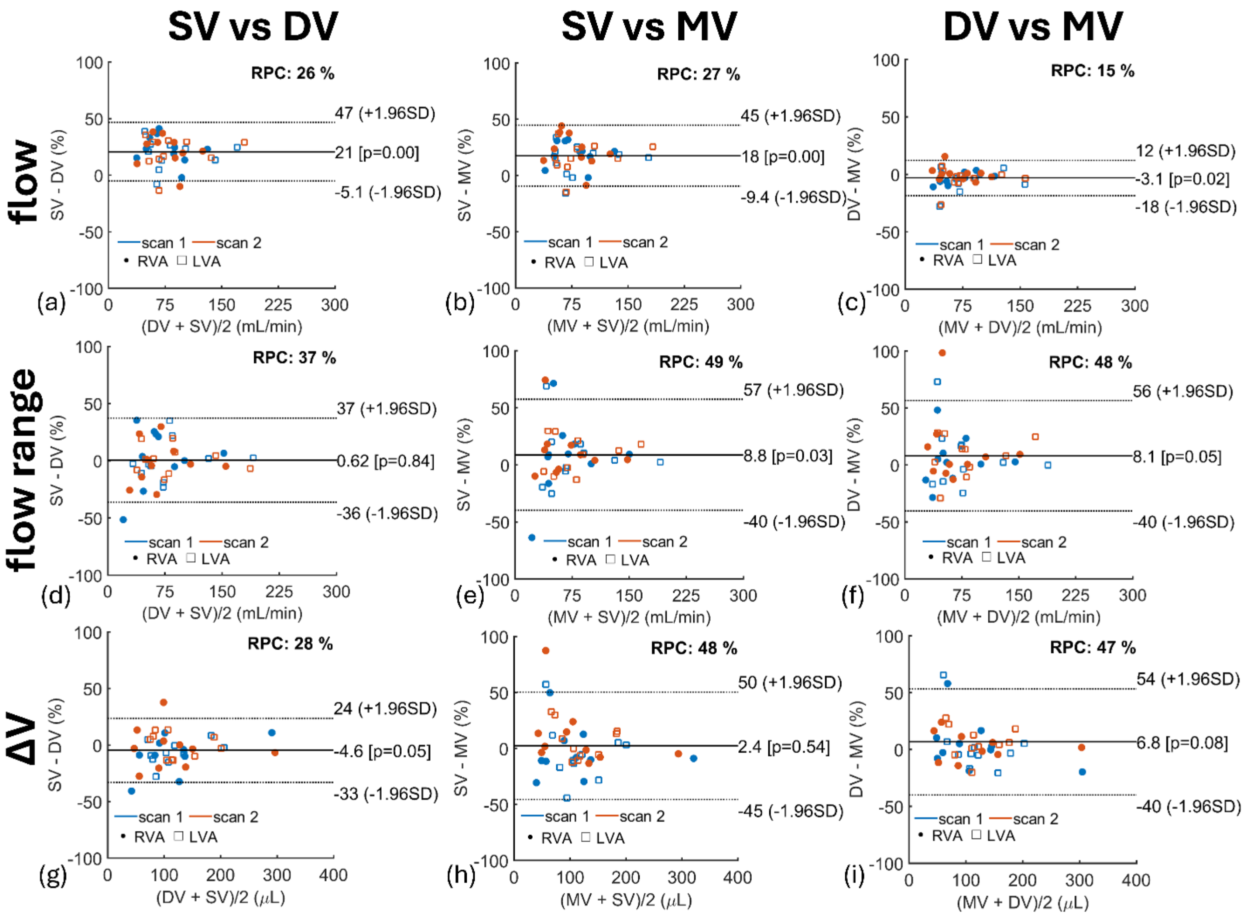

Supplemental Figure 7: Bland-Altman (BA) quantifying the effects of 2D PC flow encoding scheme on vertebral arteries (RVA, LVA) blood flow mean (top row), range (middle row) and volume change (ΔV) (bottom row) in 10 participants including repeated scans. Overall, mean flow was significantly higher in SV compared to DV and MV scans, and lower in DV compared to MV scans. Higher repeatability from repeatability coefficients (RPC) was measured for flow (a, b, c avg. RPC = 23%), compared flow range (d, e, f avg. RPC = 45%) and ΔV (g, h, i avg. RPC = 41%). MV scans VAs flow range was statistically lower compared with SV scans.

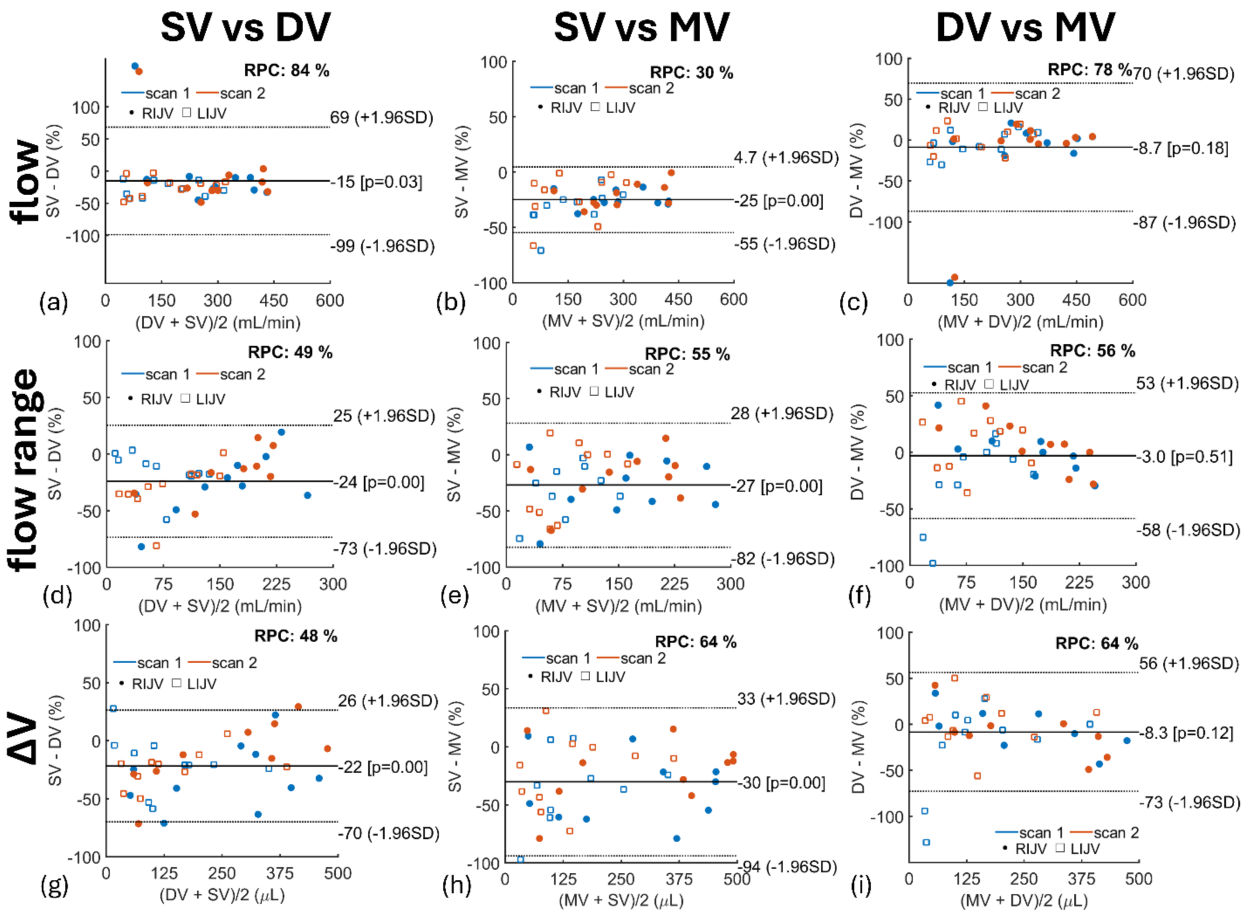

Supplemental Figure 8: Bland-Altman (BA) quantifying the effects of 2D PC flow encoding scheme on internal jugular veins (RIJV, LIJV) blood flow mean (top row), range (middle row) and volume change (ΔV) (bottom row) in 10 participants including repeated scans. Overall, blood flow means, range, and ΔV were significantly lower in SV compared to DV, and MV scans. Generally, IJVs (venous) measures had lower agreement between different flow encoding scans compared to arterial (Figure 8, Supplemental Figure 7) and CSF (Figure 7) measures. Notably, presence of uncorrectable velocity aliasing in the IJVs of one participant DV scans led to a much lower mean flow agreement with SV (a) and MV (c) scans.

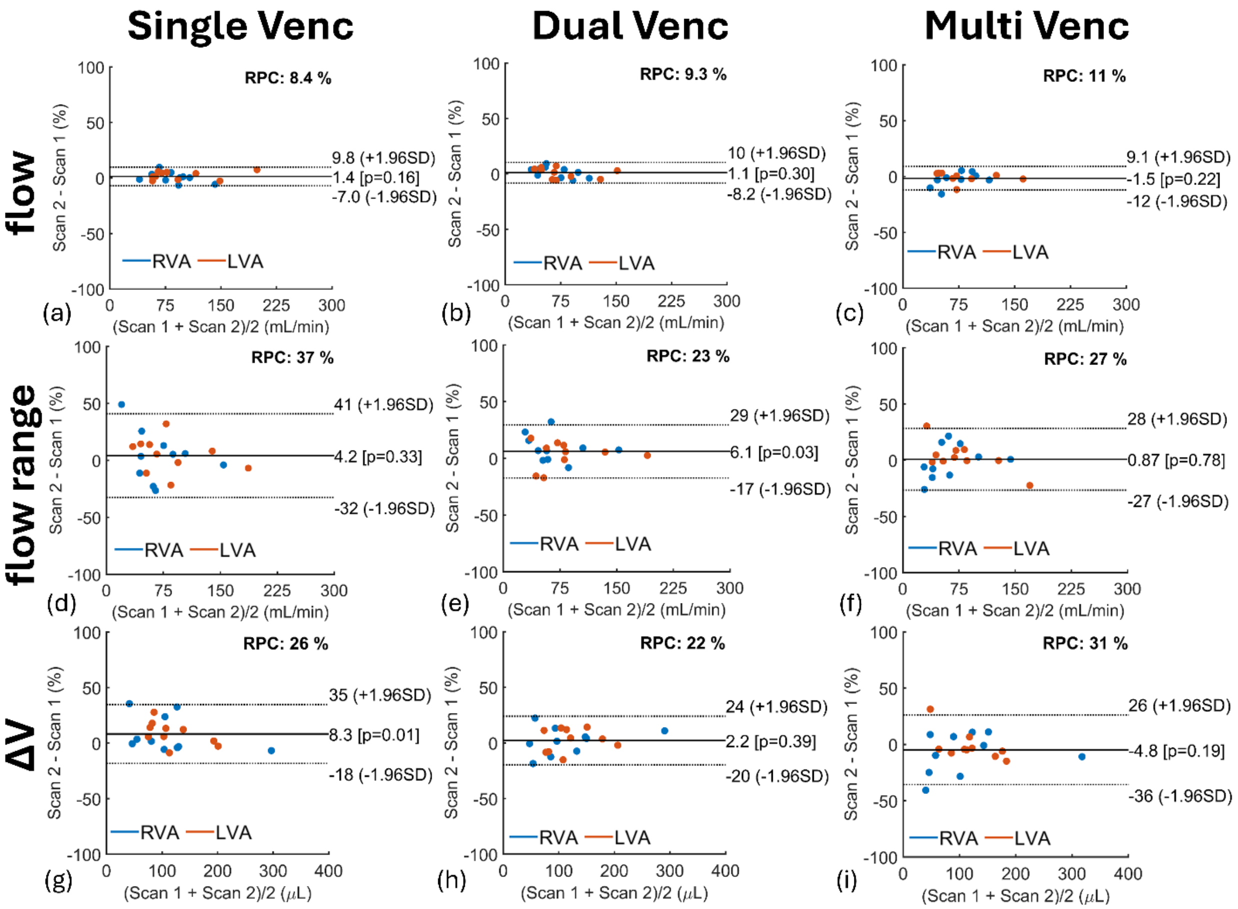

Supplemental Figure 9: Bland-Altman quantifying repeatability of 2D PC flow encoding schemes on vertebral arteries (RVA, LVA) blood flow mean (top row), range (middle row) and volume change (ΔV) (bottom row) in 10 participants. Each scan was repeated twice during the MRI study visit. The order of the acquisitions was single venc (SV) scan 1, dual venc (DV) scans 1 and 2, SV scan 2, and multi venc (MV) scans 1 and 2. Overall no significant bias were observed across repeated measures, except for flow range from DV scans and ΔV from SV scans.

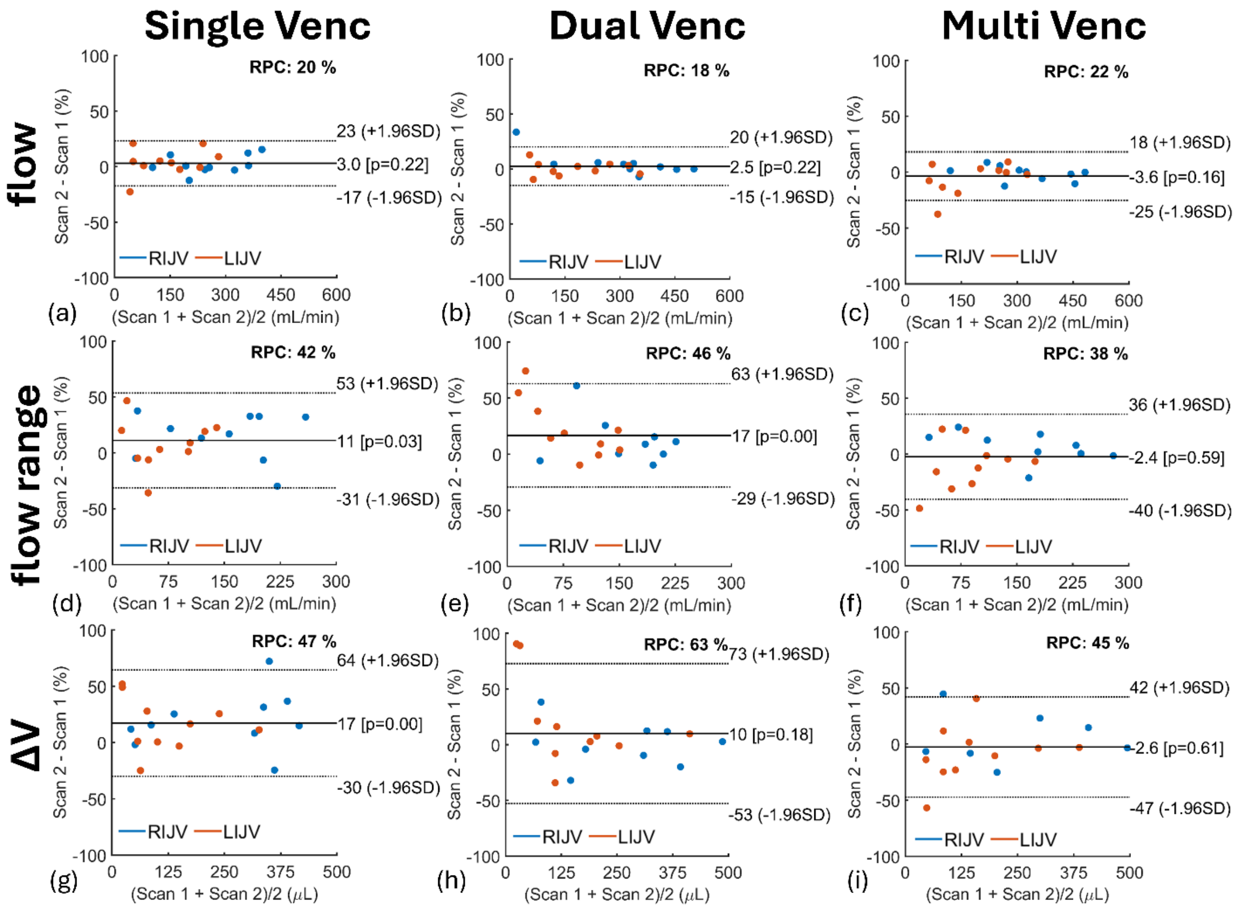

Supplemental Figure 10: Bland-Altman quantifying repeatability of 2D PC flow encoding schemes on internal jugular veins (RIJV, LIJV) blood flow mean (top row), range (middle row) and volume change (ΔV) (bottom row) in 10 participants. Each scan was repeated twice during the MRI study visit. The order of the acquisitions was single venc (SV) scan 1, dual venc (DV) scans 1 and 2, SV scan 2, and multi venc (MV) scans 1 and 2. Overall no significant bias were observed across repeated measures, except for the flow range of the DV measures (scan 2 higher flow range than scan 1), and flow range and ΔV from SV scans. Pulsatility related time-resolved markers (flow range and ΔV) generally showed lower repeatability (from higher repeatability coefficient value) compared to time-average (mean flow) measures. This is likely indicative of the noisier nature of time-resolved measurements and small pulsations of the veins compared to arteries.
